# RNA m^6^A modification regulates L1 retrotransposons in human spermatogonial stem cell differentiation in vitro and in vivo

**DOI:** 10.1007/s00018-024-05119-0

**Published:** 2024-02-16

**Authors:** Zili Li, Fang Fang, Mohammad Ishraq Zafar, Xunwei Wu, Xinyu Liu, Xia Tan, Jingwen Luo, Zhen Ye, Chengliang Xiong, Honggang Li

**Affiliations:** 1 Institute of Reproductive Health, Tongji Medical College, Huazhong University of Science and Technology, 13 Hangkong Road, Wuhan, 430030 China; 2 Department of Obstetrics and Gynecology, Union Hospital, Tongji Medical College, Huazhong University of Science and Technology, Wuhan, 430022 China; 3 Wuhan Huake Reproductive Hospital, 128 Sanyang Road, Wuhan, 430013 China; 4 Center of Reproductive Medicine, Fourth Affiliated Hospital, Zhejiang University School of Medicine, N1 Shangcheng Avenue, Yiwu, China; 5 Hubei Engineering Research Center for Preparation, Application and Preservation of Human Stem Cells, Wuhan, 430013 China

**Keywords:** m^6^A Modification, METTL3, LINE1 retrotransposon, Human germline development

## Abstract

The maintenance of genome integrity in the germline is crucial for mammalian development. Long interspersed element type 1 (LINE-1, L1) is a mobile genetic element that makes up about 17% of the human genome and poses a threat to genome integrity. N6-methyl-adenosine (m^6^A) plays an essential role in regulating various biological processes. However, the function of m^6^A modification in L1 retrotransposons and human germline development remains largely unknown. Here we knocked out the m^6^A methyltransferase METTL3 or the m^6^A reader YTHDF2 in human embryonic stem cells (hESCs) and discovered that METTL3 and YTHDF2 are crucial for inducing human spermatogonial stem cells (hSSCs) from hESCs in vitro. The removal of METTL3 or YTHDF2 resulted in increased L1 retrotransposition and reduced the efficiency of SSC differentiation in vitro. Further analysis showed that YTHDF2 recognizes the METTL3-catalyzed m^6^A modification of L1 retrotransposons and degrades L1 mRNA through autophagy, thereby blocking L1 retrotransposition. Moreover, the study confirmed that m^6^A modification in human fetal germ cells promotes the degradation of L1 retrotransposon RNA, preventing the insertion of new L1 retrotransposons into the genome. Interestingly, L1 retrotransposon RNA was highly expressed while *METTL3* was significantly downregulated in the seminal plasma of azoospermic patients with meiotic arrest compared to males with normal fertility. Additionally, we identified some potentially pathogenic variants in m^6^A-related genes in azoospermic men with meiotic arrest. In summary, our study suggests that m^6^A modification serves as a guardian of genome stability during human germline development and provides novel insights into the function and regulatory mechanisms of m^6^A modification in restricting L1 retrotransposition.

**Supplementary Information:**

The online version contains supplementary material available at 10.1007/s00018-024-05119-0.

## Introduction

The N6-methyl-adenosine (m^6^A) is the most prevalent mRNA modification in eukaryotes [1], which is catalyzed by the RNA methyltransferase complex containing methyltransferases like 3 (METTL3), METTL14, and other cofactors such as WTAP, VIRMA, and ZC3H13 [2–4]. This modification can be erased by demethylases FTO and ALKBH5 [5, 6]. Most m^6^A sites are found in the conserved motif DRACH (D = G/A/U, R = G/A, H = A/U/C), which is frequently located around the transcription start site (TSS) region and the stop codon through whole-transcriptome m^6^A sequences [7]. It has been reported that m^6^A modification regulates gene expression through multiple mechanisms, including pre-mRNA splicing, mRNA export, stability, translation, and modulation of the binding capacity of m^6^A binding "reader" proteins such as YTHDF1-3, YTHDC1-2, and IGF2BP1-3 [8]. Therefore, by mediating a series of RNA processing steps, m^6^A modification regulates various cellular physiological and pathological processes, such as heat shock response, cell reprogramming, circadian rhythm, hematopoiesis, and tumorigenesis [9, 10].

Approximately one-third to half of the typical mammalian genome is derived from transposable elements (TEs) [11]. The TEs are mobile genetic elements that can insert themselves into new locations in the genome, giving rise to repeated copies of the elements and contributing an abundance of genetic novelty to host genomes, including unique protein-coding genes and cis-acting regulatory elements [12]. Long interspersed element type 1 (LINE-1, L1), active for around 160–190 million years, is the most successful TE family across therian mammals, comprising about 17% of the human genome [13]. L1 elements are about 6 kb long and encode three open reading frames, encoding a newly identified ORF0 with an unknown function, L1 RNA-binding protein ORF1p, and ORF2p with endonuclease and reverse transcriptase activities. These activities are required for inserting L1 cDNA into target DNA, which induces DNA double-strand breaks (DSBs)[14].

The host cells evoke several defense mechanisms to protect themselves against deleterious retrotransposition, including DNA methylation, PIWI-interacting RNA (piRNA)-PIWI systems, microRNAs, and host restriction factors, such as APOBEC3, MOV10, and MAVS [15]. Recent studies on mouse embryonic stem cells (mESCs) have shown that METTL3-METTL14 and FTO-mediated m^6^A RNA methylation acts to restrict endogenous retroviruses (ERVs), especially L1 RNA and intracisternal A-particles (IAPs), by recruiting the YTHDF family to target their 5′ untranslated region [16, 17]. Additionally, m^6^A binding protein YTHDC1 facilitates the decay of a subset of m^6^A-modified RNAs, especially L1 elements, through NEXT-mediated nuclear degradation [18]. Moreover, a study on human cancer cells identified that METTL3 facilitates L1 retrotransposition, while m^6^A demethylases ALKBH5 suppress it, thereby allowing L1 retrotransposons to hijack the RNA m^6^A modification system for successful replication in HeLa cells [19]. Furthermore, the evolutionarily young L1s are prominently marked by m^6^A modification in K562 cells and lead to promoting L1 RNA expression and retrotransposition [20]. These discrepancies between humans and mice might be due to the different cell lines (cancer cells versus ESCs) or species.

Here, we have found that inactivating METTL3 in hESC facilitates L1 retrotransposition in SSCs induction in vitro, and L1 mRNA is degraded by autophagy through binding to YTHDF2, consequently blocking L1 retrotransposition. By analyzing human fetal testis, we discovered that m^6^A modification in human fetal germ cells promotes L1 retrotransposon RNA degradation, preventing the insertion of new L1 retrotransposon into the genome. Moreover, L1 retrotransposon RNA expression is higher, whereas *METTL3* is downregulated in the seminal plasma of azoospermic patients with meiotic arrest, compared to males with normal fertility. Analysis of whole exome sequencing (WES) data from meiotic arrest patients has also found potentially pathogenic variants in m^6^A-related genes. Thus, our data presents a new foundation for understanding the biological roles of m^6^A modification and L1 retrotransposon in human germ cell development and has broad implications for the mechanism of male infertility, especially for nonobstructive azoospermia.

## Materials and methods

### hESC culture

The hESCs were maintained in a feeder-free condition using mTeSR1 (Stem Cell Technologies, 85850) on Matrigel (Corning, 356234)-coated cell culture plates. Cultures were passaged at a 1:10–1:15 split ratio every 4–5 days using 0.5 mM EDTA/PBS (Thermo Fisher, AM9260G). A 10 μM Rho-associated protein kinase (ROCK) inhibitor, Y-27632 (Selleck Chemicals, S1049), was added to the culture medium during cell passaging or thawing.

### Generation of mutant lines

CRISPR gRNAs for *METTL3* and *YTHDF2* are supplied in Supplementary Tables, and these gRNAs were cloned into pX330 (Adegene, #42230). For transfection, 80% of confluent cells were dissociated into single cells using Accutase (Thermo Fisher, 00-4555-56). 1 × 10^6^ cells were then plated in a Matrigel-coated 35-mm dish and transfected with 1 μg of the CRISPR plasmid in 100 μl of OptiMEM (Thermo Fisher, 31985088) using 3 μl of FuGENE 6 (Promega, E2691). 2 μM Thiazovivin (Selleck Chemicals, S1459) was added in culture medium to improve survival rates of cells. Another transfection was performed 24 h later to improve the transfection rate. 24 h after the two rounds of transfection, hESCs were dissociated into single cells and re-plated at 300 cells per 10-cm dish, and CloneR (Stem Cell Technologies, 05889) was added to the culture medium. 7 ~ 9 days later, the single cell-derived colonies were dissociated by Collagenase IV (Stem Cell Technologies, 07909) and manually picked individually into 48-well plates for amplification. Colonies were analyzed by Sanger sequencing at the *METTL3* and *YTHDF2* genes for the presence of mutations. The cell pellets were suspended in 20 μl of water, and 2 μl of the suspended cells were used for PCR amplification of the target site with the KOD FX (TOYOBO, KFX-101) kit according to the manufacturer’s protocol. The primers are listed in Supplementary Tables. Clonal cell lines carrying the desired mutations were amplified and frozen. WT cell lines were used as passage-matched controls for analysis and differentiation.

### Induction of hPGCLCs

The hESCs were dissociated with 0.5 mM EDTA/PBS, and 1 × 10^6^ cells per well were plated on a Matrigel-coated 35 mm dish in GK15 medium (G-MEM [Thermo Fisher, 11710-035], 15% KSR [Thermo Fisher, 10828-028], 0.1 mM NEAA [Thermo Fisher, 11140-050], 2 mM L-glutamine [Thermo Fisher, 35050-061], 1 mM sodium pyruvate [Thermo Fisher, 11360-070], 0.1 mM 2-mercaptoethanol [Sigma, M3148], 3 μM CHIR99021 [Selleck Chemicals, S2745], 50 ng/ml Activin A [PEPRO TECH, 120-14E] and 10 μM ROCK inhibitor) for pre-induction. After 40 ~ 42 h of pre-induction, the cells were dissociated with Accutase (Thermo Fisher, A1110501) and plated into ultra-low cell attachment U-bottom 96-well plates (Corning, 7007) at a density of 2,000–4,000 cells per well to form embryoid bodies in 200 μl of aRB27 induction medium (Advanced RPMI 1640 [Thermo Fisher, 126330012], 1% B-27 supplement [Thermo Fisher, 175040044], 0.1 mM NEAA, 2 mM L-glutamine, 500 ng/ml BMP4 (R&D Systems, 314-BP-050), 10 ng/ml human LIF (R&D Systems, 7734-LF-100), 100 ng/ml SCF (R&D Systems, 255-SC-050), 50 ng/ml EGF (R&D Systems, 236-EG-200), and 10 μM ROCK inhibitor (Selleck, S1049).

### Induction of SSCLCs

hESCs were seeded onto Matrigel-coated 24-well plates in mTeSR1 medium containing 10 μM ROCK inhibitor and the medium was replaced by an SSCLC induction medium on the second day. The components of the SSCLC induction medium contained α-MEM, 3% KSR, 1% GlutaMAX supplement, 1% 100 × Insulin Transferrin Selenium X (Thermo Fisher, 51500056), 0.2% chemically defined lipid concentrate (Thermo Fisher, 11905031), 20 ng/mL human GDNF (R&D Systems, 212-GD), 1 ng/mL human b-FGF (R&D Systems, PHG0266), 100 μg/mL Valproic acid (Selleck, S3944), and 100 μg/mL Vitamin C (Selleck, S3114). The SSCLC induction medium was changed daily until the cells were ready for analysis.

### Flow cytometry analysis

To analyze hPGCLCs or hESCs using cell surface markers, we stained the dissociated cells with CD24, CD90, EpCAM, and INTEGRINα6. Intracellular staining was performed using a BD kit (BD, 560589) according to the manufacturer’s instructions. For analysis, SSCLCs cells were dissociated into single cells using Accutase and fixed with 4% paraformaldehyde for 15 min at room temperature. After blocking and permeabilizing, the cells were suspended in 100μL of PBS containing 0.1% BSA and stained with a PLZF monoclonal antibody-PE (Invitrogen, 12-9320-82) for 40 min at room temperature. The cells were then washed, resuspended in PBS, and the percentage of PLZF-positive cells was detected by a DxFLEX flow cytometer (Beckman Coulter). The primary antibodies used in this study are listed in Supplementary Tables.

### Quantitative RT-PCR

The total RNA was extracted using TRIzol reagent (Thermo Fisher, AM9738) or MicroElute Total RNA Kit (OMEGA, R6831-01). The cDNA was synthesized using the Reverse Transcription Kit (Takara, RR047A). The qRT-PCR was performed using SYBR Premix Ex Taq II (Takara). The primers used are shown in the Supplementary Tables. The values were normalized to GAPDH or β-actin and shown relative to control samples. The error bars represent the mean ± s.d. from three independent experiments.

### Cell proliferation

Cell viability was detected by adding CCK8 (Abcam, ab228554) to cells plated in 96-well plates, followed by incubation at 37 °C for 1–2 h on days 0–6. The absorbance of each well was measured using a microplate reader set at 460 nm.

### Human sample collection and processing

Human fetal specimens were collected without patient identifiers after elective termination of pregnancy. All fetal samples were transported on ice for gonad microdissection. Human adult nonobstructive azoospermia seminal plasma was collected free of the patient before microscopic testicular sperm extraction (microTESE). Specimens were either flash-frozen for molecular biology analysis or fixed for immunofluorescent staining. For staining, tissues were fixed overnight in a 4% PFA in PBS solution at 4 °C. The tissues were then washed, incubated in 10%, 20% and 30% sucrose at 4 °C, embedded, and frozen in OCT solution (Tissue-Tek, 4583). The embedded tissues were cryosectioned at 8 μm.

### Immunofluorescence

The cells were fixed in 4% paraformaldehyde at room temperature for 15 min. After washing and permeabilization for 10 min with Wash Buffer (0.01% Triton X-100 and 1.0% BSA in PBS), the cells were incubated with primary antibodies in Wash Buffer at 4 °C overnight. Then the cells were washed three times and incubated with secondary antibodies at room temperature for one hour. After washing three times, the cells were incubated with DAPI for 5 min at room temperature, and images were captured using a confocal microscope (Leica) and processed with Leica software. To detect L1 RNA, RNAscope Probe-Hs-LINE1-ORF1p-C1 (bio-techne, 1075751-C1) was used and operated according to the manufacturer’s instructions. The corresponding negative (bio-techne, 320871) and positive (bio-techne, 320861) control probes were added. The primary antibodies used are listed in Supplementary Tables.

### Plasmid construction

The sequence of human *METTL3* cDNA was amplified from pENTR-METTL3 (Vigene Bioscience, CH8066235). The PCR products were first cloned into pENTR1A no ccDB (Adegene, #17398) by Gibson Assembly Mix (NEB, #E2611) according to the manufacturer’s protocol. The cloned products were then recombined into the pLEX_307 (Addgene, #41392) using the LR Clonase Enzyme Mix (Thermo Fisher, 11791020). The METTL3 mutant plasmid was constructed by Mut Express II Fast Mutagenesis Kit (Vazyme, C214-01) according to the manufacturer’s protocol. All sgRNA was cloned into the px330 vector (Addgene, #42230), sgRNAs, and primers used for the construction are shown in Supplementary Tables.

1 × 10^6^ hESCs were infected with the lentiviruses. 0.25 μg/ml puromycin (Thermo Fisher, A1113803) was added 7 ~ 10 days after the infection.

### LINE-1 retrotransposition assay

The pBS-L1PA1-CH-mneo constructs lacks a 5′ UTR. To create a 5′ UTR-containing pBS-L1PA1-CH-mneo construct, the 5′ UTR of L1Hs was amplified using PCR and inserted downstream of the CMV promoter of pBS-L1PA1-CH-mneo by Gibson clone kit (NEB, E2611). Retrotransposition assays were performed as described before [20]. Briefly, 1 × 10^6^ day6 SSCLCs were plated in a Matrigel-coated 35-mm dish and transfected with 1 μg of the pBS-L1PA1-CH-mneo plasmid in 100 μl of OptiMEM using 3 μl of FuGENE 6. 2 μM Thiazovivin was added in culture medium to improve survival rates of cells. Another transfection was performed 24 h later to improve the transfection rate. Two days later, 100 μg/ml of Geneticin (Thermo Fischer, 10131027) was added to the SSC induction media to select the transfected cells. Cell selection continued for 7–10 days, and the colonies were then stained with crystal violet and counted.

### MeIP-qPCR and RIP-qPCR

MeIP-qPCR and RIP-qPCR were performed using the Magna MeRIP m^6^A Kit (Merck, 17-10499) and Magna RIP RNA-Binding Protein Immunoprecipitation Kit (Merck, 17-701) according to the manufacturer’s protocols. The antibodies and primers used for RIP and qPCR are provided in Supplementary Tables.

### Western blot

Whole-cell extracts were in lysis buffer composed of 50 mM Tris–HCl (pH 7.5), 0.15 M NaCl, 0.1% SDS, 1% Triton X-100, 1% sodium deoxycholate, and protease inhibitor cocktail (Sangon Biotech, C600386). After electrophoresis, proteins were transferred to nitrocellulose membranes. The membranes were incubated in a western blocking reagent (TBST with 5% non-fat milk) for one hour at room temperature. Antibodies, diluted in blocking buffer, were added to the membranes and incubated overnight at 4 °C. The membranes were washed three times for 15 min each in TBST, and then incubated with a secondary antibody in blocking buffer for one hour at room temperature. The membrane was washed three times in TBST. Thermo Scientific Pierce ECL Western Substrate (Thermofisher, 32106) was used for detection. The original western blot results can be found in the supplemental material.

### ***m***^***6***^***A dot blot***

mRNA was enriched using VAHTS mRNA Capture Beads (Vazyme, #61006) from total RNA. mRNA samples in a volume of 2 µL were denatured by heating at 72 °C for 5 min and chilling on ice to prevent secondary structure remodeling. Then, mRNA was loaded on the Amersham N + membrane (GE Healthcare, #RPN303B) and crosslinked to the membrane by UV and detected with an m^6^A-specific antibody (ABclonal, A19841). Another membrane was stained with methylene blue as a loading control.

### ***m***^***6***^***A ELISA***

m^6^A ELISA was performed using EpiQuik m^6^A RNA Methylation Quantification Kit (EpigenTek, P-9005-48) according to the manufacturer’s protocol, and 200 ng of mRNA was used. Absorbance was measured at 450 nm within 5 min using an absorbance microplate reader.

### RNA decay with actinomycin D

Dimethylsulphoxide or Bafilomycin (20 nM, Selleck, S1413) was added to day 10 SSCLCs for 1 h, then Actinomycin D (5 μg/ml, Selleck, S8964) was added for 6 h. RNA was extracted from cells immediately using TRIzol reagent (Thermo Fisher, AM9738), and cDNA was synthesized using Reverse Transcription Kit (Takara, RR047A). qRT-PCR was performed using SYBR Premix Ex Taq II (Takara).

### RNA-seq analysis

Before mapping, reads were quality-trimmed (Q > 25), and the adaptor sequence was removed using Trim-Galore v0.6.4. Reads were mapped to the human reference genome (GRCh38/hg38) by HISAT2 v7.5.0 reporting randomly one position. Read counts were derived from the feature Counts v2.0.1 with default parameters and retrotransposon annotations bed files (RepeatMasker) were downloaded from the UCSC table browser. The R Bioconductor DESeq2 package v1.28.1 was used to normalize counts per Ref Seq transcripts to evaluate the differential expression. Before clustering and principal component analysis of 10 × genomic data, the transcripts with the 10% lowest average expression were removed, and the gene expression data matrix was centered and scaled. Principal component analysis was performed by the R Bioconductor Seurat package v3.2.0. Gene ontology analysis was performed by the R Bioconductor ClusterProfiler package v3.16.1.

### MeIP-seq

MeRIP-seq was carried out using the Magna MeRIP m^6^A Kit (Merck, 17-10499) according to the manufacturer’s instructions. In short, total RNA was extracted using Trizol. 350 μg of genome DNA was erased and chemically fragmented total RNA was subjected to immunoprecipitation with affinity-purified anti-m^6^A antibody in the presence of RNase inhibitor. Bound m^6^A-methylated RNA fragments were eluted and purified using the RNeazy Kit (Qiagen) and processed for library generation using the SMARTer Stranded Total RNA-Seq Kit (TaKaRa, 634411), following the manufacturer’s recommendations, but without fragmentation step. Sequencing was performed using the Illumina Novaseq 6000. Input for each cell line was sequenced as a control. Reads were mapped to the human reference genome (GRCh38/hg38) by Bowie2 v2.4.5. Peaks enriched in the MeRIP sample over the input control were defined using MACS2 v2.2.7.1.

### RIP-seq

RIP-seq was performed using the EZ-Magna RIP RNA-Binding Protein Immunoprecipitation Kit (Merck, 17-701) according to the manufacturer’s protocols. In short, total RNA was extracted using Trizol (Life Technologies). cDNA libraries were produced by employing a NEBNext UltraRNA Library Prep Kit for Illumina (New England Biolabs) and sequenced on an Illumina NovaSeq 6000 platform at Seqhealth Ltd. (Wuhan, China) following the vendor’s recommended protocol.

### Whole exome sequencing

WES was performed by Wuhan SeqHealth company. The quality of the raw data was evaluated using the FastQC packages. Reads were aligned to the reference human genome GRCh38 using bowtie package. SNVs and small indels were called using freeBayes v0.9.21 (Speedseq-var). Variant files obtained from the whole genome data were annotated using Ensembl Variant Effect Predictor.

### Statistical analysis

Data are presented as means ± s.d. and were derived from at least three independent experiments. Data on replicates (n) are given in Figure legends. Statistical analysis was performed using the two-sided Student’s t-test (comparing two groups) or one-way-comparison ANOVA (comparing multiple groups against one group).

## Results

### METTL3 is essential for human spermatogonial stem cell differentiation during spermatogenesis

**Fig. 1:**
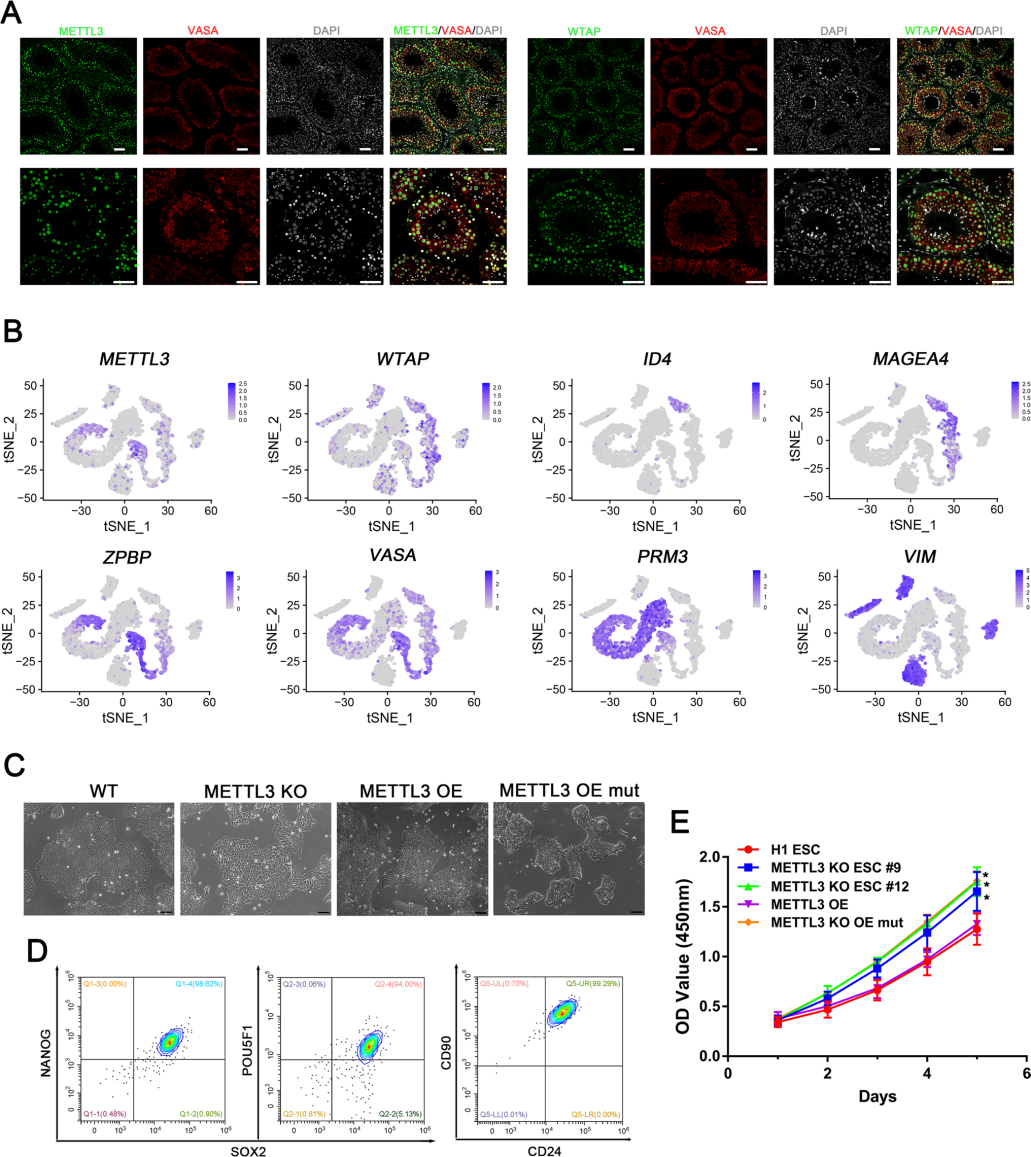
METTL3 is expressed in human germ cells and inactivated in hESCs. **A** Immunofluorescence images (adult human testicular tubule cross-section) stained with METTL3, WTAP, and VASA, Scale bar = 50 μm; **B** Dimension reduction representation of single-cell RNA-seq (t-SNE, t-Distributed Stochastic Neighbor Embedding) measured in adult human testis, showing m^6^A methyltransferase complex *METTL3* and *WTAP* co-expressed with *VASA* in germ cells. *ID4* represents spermatogonial stem cells, *MAGEA4* represents spermatogonia, *ZPBP* represents spermatocytes, *PRM3* represents spermatids, and *VIM* represents somatic cells; **C** Bright field images of WT, METTL3 KO, wild type METTL3 overexpression, and mutant METTL3 overexpression in hESCs, Scale bar = 100 μm; **D** FACS analysis of POU5F1, SOX2, NANOG, CD24, and CD90 expression in METTL3 KO hESCs; **E** Growth curves of WT, METTL3 KO, wild type METTL3 overexpression and mutant METTL3 overexpression hESCs; n = 3 independent experiments. Data are presented as means ± s.d. Statistical analysis was performed by Student’s *t-*test (two-sided), *Represents compared to the H1 ESC group *p* < 0.05

To explore the role of m^6^A modification in human spermatogenesis, we conducted an immunostaining assay to determine the expression of two m^6^A methyltransferase complexes, METTL3 and WTAP, in the human testis. Our findings revealed that both proteins were localized in the nucleus of male germ cells and somatic cells (Fig. 1A). We then analyzed published data based on 10 × genomics single-cell sequences of human testes [21] and found that the core methyltransferase complexes, *METTL3* and *WTAP*, are mainly expressed in germ cells (marked with *VASA*). Notably, WTAP is predominantly co-expressed with spermatogonial stem cells (marked with *ID4*) and spermatogonia (marked with *MAGEA4*), whereas METTL3 is primarily co-expressed with spermatocytes (marked with *ZPBP*) and a portion of spermatids (marked with *PRM3*). Somatic cells (marked with *VIM*) exhibit minimal expression of *METTL3* and *WTAP* (Fig. 1B). Overall, these results suggest that m^6^A modification plays a crucial role in spermatogenesis.

To further investigate the function of METTL3-mediated m^6^A modification in early human germ cell development, we specifically used the CRISPR-Cas9 system to knockout the *METTL3* gene in hESCs. The knockout cells were analyzed for targeted mutations of the relevant loci through DNA sequencing and western blotting (Fig. S1A, B). We did not find any alternatively spliced METTL3 transcript isoforms around 50 kDa [22]. However, the depletion of METTL3 resulted in the loss of colony structural integrity, as indicated by poorly defined edges in Fig. 1C. Despite this, pluripotency markers such as NANOG, SOX2, and POU5F1 (also known as OCT4) were still expressed in the METTL3 KO hESCs as shown in Fig. 1D and Fig. S1E. Moreover, METTL3 KO hESCs showed a higher proliferation rate compared to WT hESCs (Fig. 1E). The enzyme-linked immunosorbent assay (ELISA) and m^6^A dot blot showed a significant reduction of m^6^A RNA methylation levels in the METTL3 KO hESCs (Fig. S1C, D). Notably, the overexpression of wild type METTL3, but not the catalytically compromised METTL3 (DPPW motif mutated to APPA, Fig. S1F), in the METTL3 knockout cells rescued m^6^A RNA methylation levels and colony structural integrity (Fig. 1C, Fig. S1C, D).

Firstly, we utilized our optimized method to generate human primordial germ cells (hPGCs) [23, 24] from hESCs. However, there was no difference in the percentage of EpACM/INTEGRINα6 double-positive hPGCLCs between METTL3 KO hESCs and WT hESCs, as determined by FACS (Fig. S2A, B). Furthermore, the METTL3 KO cells expressed essential PGC genes such as *SOX17*, *TFAP2C*, *NANOS3*, *BLIMP1*, and *POU5F1* upon hPGC induction (Fig. S2C), suggesting that the METTL3 protein is dispensable for the initiation of hPGC specification. Additionally, the expression of *SOX2*, which is upregulated in mPGCs, was downregulated in both METTL3 KO and WT hPGCLCs (Fig. S1C). These results differ from the mouse model in which early Oct4/Blimp1 double-positive PGCs were not induced in Mettl3 KO post-implantation mouse embryos [25].

**Fig. 2:**
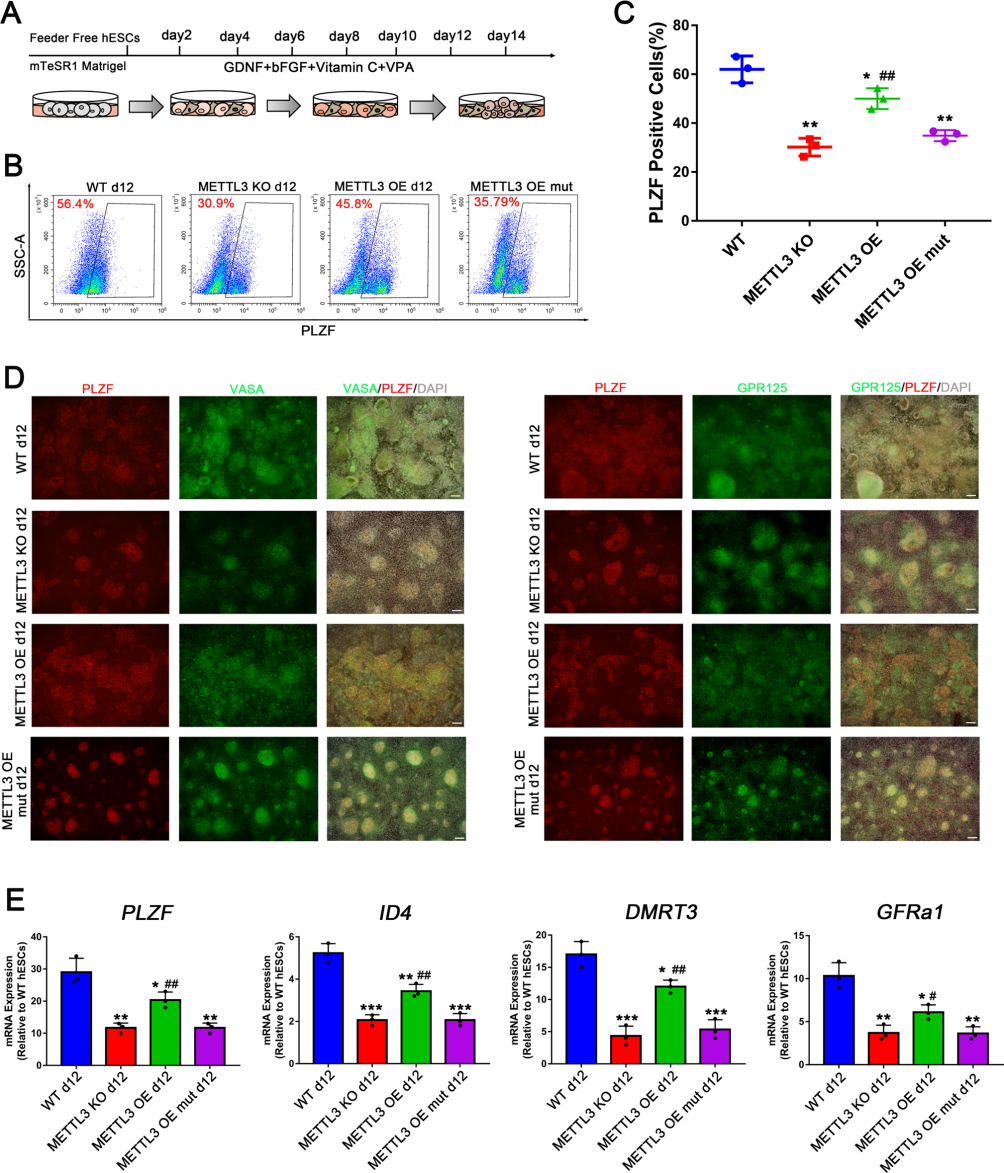
METTL3 is essential for hSSCLCs differentiation. **A** Scheme of hSSCLCs differentiation in vitro; **B** FACS analysis of WT and METTL3 KO hESCs on hSSCLCs induction for 12 days. Boxed areas indicate the percentages of PLZF positive cell; **C** Quantification of FACS data at day 12 of hSSCLCs induction for each cell type; n = 3 independent experiments. Data are presented as means ± s.d. Statistical analysis was performed by one-way ANOVA: *p < 0.05, **p < 0.01 indicate a significant difference compared to the WT group; ## *p* < 0.01 indicates a significant difference compared to the METTL3 KO group; **D** Immunofluorescence staining of PLZF, VASA, and GPR125 at day 12 of each cell line. Scale bar = 100 μm; (E) RT-qPCR analysis of gene expression during hSSCLCs differentiation on day 12; n = 3 independent experiments. Data are presented as means ± s.d. Statistical analysis was performed by one-way ANOVA: *p < 0.05, **p < 0.01, ***p < 0.001 indicate a significant difference compared to the WT group; ^#^*p* < 0.05, ^##^*p* < 0.01 indicate a significant difference compared to the METTL3 KO group

**Fig. 3:**
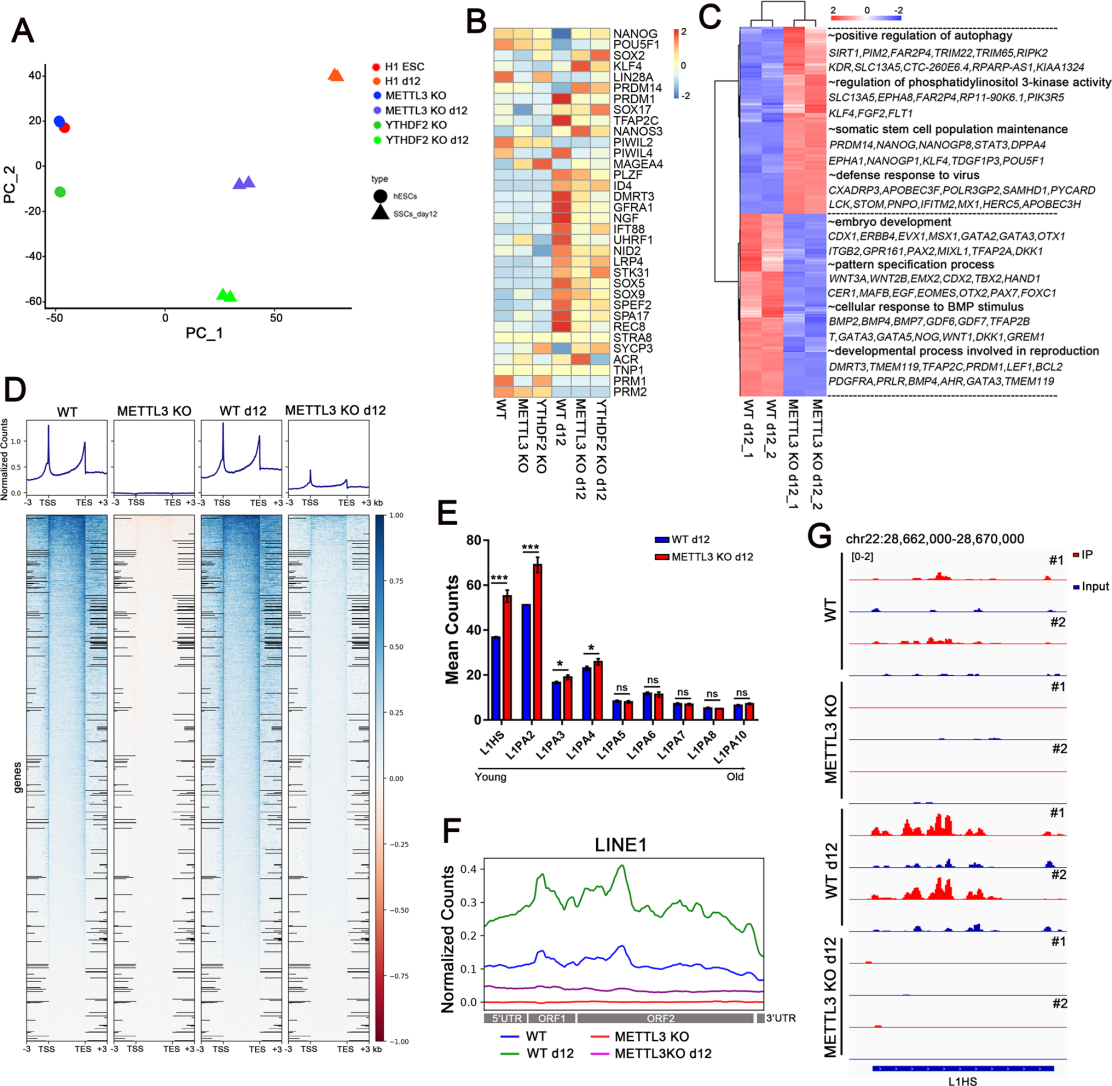
Depletion of the m^6^A methyltransferase METTL3 increases L1 retrotransposon RNA levels. **A** A PCA plot of RNA-seq data from different cell lines. Color codes indicate the cell types and shapes for cell states; **B** A heat map indicating key markers in hESCs and hSSCLCs; **C** Differentially expressed gene levels are represented by a heat map. Gene Ontology (GO) functional terms and representative genes are shown for each gene cluster; **D** The normalized MeRIP-seq read count in each cell line across the -3 kb upstream of the transcription start sites (TSS), through scaled gene bodies (5 kb) to + 3 kb downstream of transcription end sites (TES) of mRNA, n = 2 independent experiments; **E** Histogram plots showing the expression of different L1 sub-families counts in METTL3 KO day 12 hSSCLCs and WT day 12 hSSCLCs. n = 2 independent experiments. A random assignment of multi-mapped reads method was used for L1 counting. Data is expressed as means ± s.d. Statistical analysis was performed by Student’s-*t* test (two-sided), *p < 0.05, *** *p* < 0.001 indicate a significant difference compared to the WT group; **F** The normalized MeRIP-seq read count in each cell line across the 5′ UTR, ORF1, ORF2, and 3′ UTR of L1 mRNA with at least one m^6^A peak, n = 2 independent experiments; **G** A genome browser snapshot of MeRIP-seq data at the L1HS locus, n = 2 independent experiments

Next, we used our previously published method to generate human spermatogonial stem cell-like cells (hSSCLCs) [26] from hESCs. Intriguingly, about 60% of promyelocytic leukemia zinc finger (PLZF, a marker of rodent stem and progenitor spermatogonia [27]) positive hSSCLCs were induced from WT hESCs, while only 30% of the derived METTL3 KO cells were positive for PLZF (Fig. 2A–C). Moreover, the overexpression of METTL3 in METTL3 KO hESCs only partially rescued the induction of hSSCLCs (Fig. 2A–C). Immunofluorescence and RT-qPCR analyses confirmed that the expression levels of SSC markers such as PLZF, VASA, GPR125, ID4, DMRT3, and GFRa1, were consistent with the efficiency of SSC induction in the different groups (Fig. 2D, E). Additionally, the differentiated hSSCLCs from METTL3 KO cells displayed high levels of *NANOG* and *POU5F1* expression but lacked expression of SSC markers (Fig. 3B), reflecting that a considerable number of cells failed to exit the hESCs program in the mutant cells [28]. These results highlight the importance of METTL3 in human SSC differentiation, as its deletion leads to defects in spermatogonial differentiation in vitro.

### METTL3 modified L1 retrotransposons RNA in hSSCLCs differentiation

To comprehensively understand the characteristics of hESCs and induced hSSCLCs, we compared the transcriptome and m^6^A modification of WT and METTL3 KO hESCs, as well as day 12 SSCLCs using high-throughput RNA sequencing and methylated RNA immunoprecipitation sequencing (MeRIP-seq).

Principal Component Analysis (PCA) showed that most gene expressions in hESCs were not influenced by the deletion of METTL3 (Fig. 3A). Compared with WT hESCs, METTL3 KO hESCs showed upregulation of 496 genes, which were enriched in synaptic signaling and DNA packaging complex genes. The 362 down-regulated genes were significantly enriched in genes required for cell adhesion and extracellular matrix organization. This could potentially explain the presence of uncompacted colonies in METTL3 KO hESCs (Fig. S3A, B). In contrast, the transcriptome of WT and METTL3 KO day 12 SSCLCs remarkably changed. A total of 1209 genes were down-regulated in METTL3 KO day 12 SSCLCs, and gene ontology (GO) analysis showed these items were enriched in embryo and reproduction development, while the 1238 up-regulated genes were enriched in autophagy and defense responses to the virus (Fig. 3C, Fig. S3C). Given that maintenance of genome integrity and genomic stability are crucial for germline and early embryo development, we focused on the changes of transposable elements (TEs) in METTL3 KO SSCLCs. RNA sequencing (RNA-seq) analysis confirmed a significant upregulation of TEs in METTL3 mutant SSCLCs, particularly in evolutionarily young L1s such as L1HS, L1PA2/3/4 [29] (Fig. 3E, Fig. S4D). However, TEs were rarely changed between WT and METTL3 KO hESCs (Fig. 6A, Fig. S4D). MeRIP-seq results revealed that METTL3 KO cells displayed decreased m^6^A modification (Fig. 3D, F). Meanwhile, m^6^A sites were found to be enriched in the m^6^A consensus motif across all cell lines (Fig. S3E). Notably, m^6^A sites were significantly enriched near the TSS and beginning region of the 3′UTR of protein-coding mRNA, while m^6^A sites in L1 were mainly enriched in full-length L1 (Fig. 3D, F). The analyses from MeRIP-seq and m^6^A RT-qPCR confirmed a decrease in m^6^A modification on L1HS in METTL3 KO cells (Fig. 3G, Fig. S4A). Additionally, it was observed that genes containing m^6^A were more highly expressed in WT samples compared to METTL3 KO samples, while genes without m^6^A modification did not exhibit this tendency (Fig. S3D).

The immunofluorescence results indicate that PLZF positive SSCLCs in both the WT and METTL3 KO groups exhibited lower levels of L1 ORF1p (Fig. S4B). Moreover, the western blotting results support the GO analysis findings derived from RNA-seq, suggesting that m^6^A methylation regulates L1 retrotransposons in a post-transcriptional way by autophagy [30] (Fig. S4C).

### L1 retrotransposon was degraded in a YTHDF2-dependent manner

**Fig. 4:**
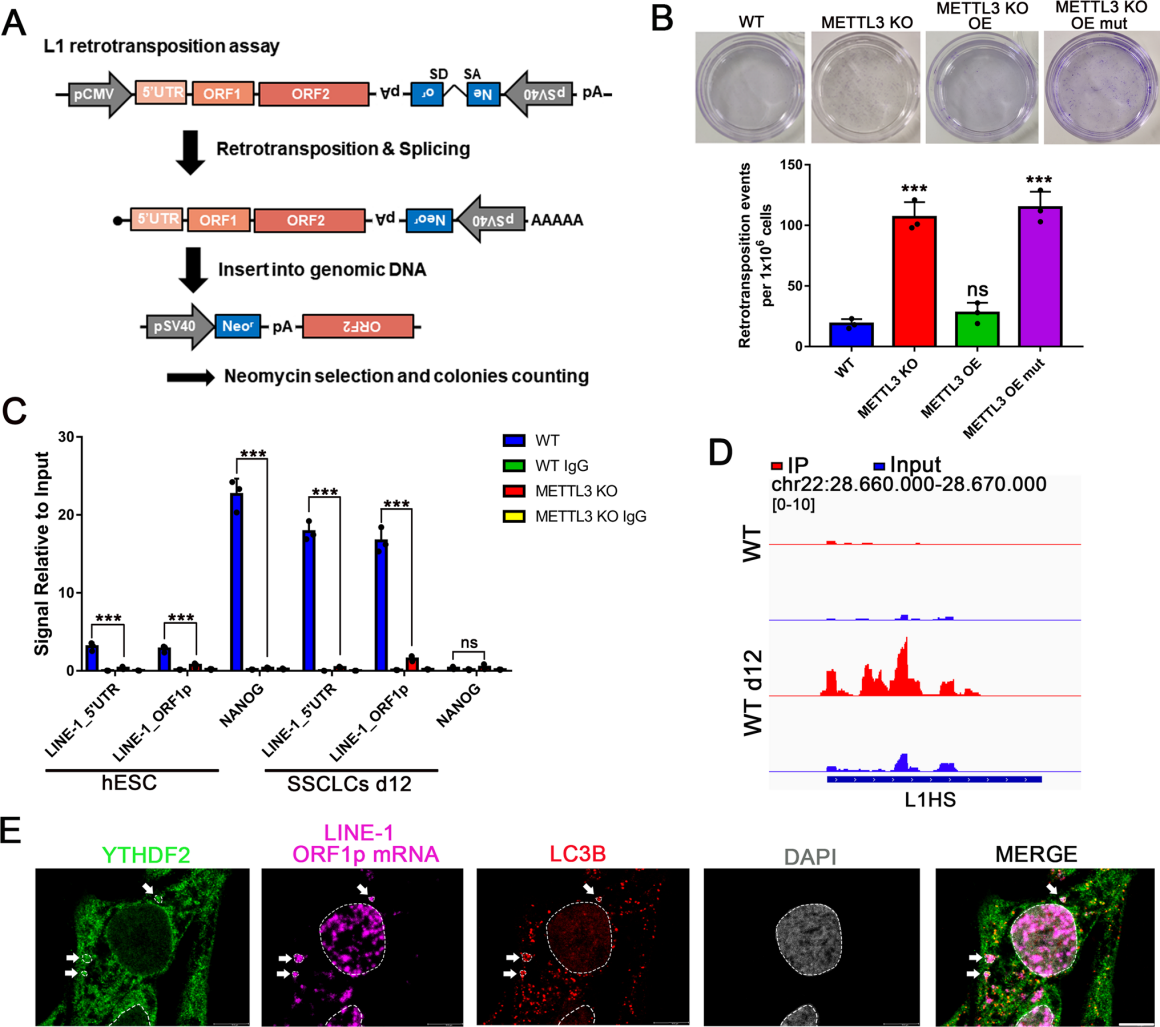
m^6^A modification regulates L1 retrotransposon degradation in a YTHDF2-dependent manner. **A** A schematic of the L1 construct and an overview of the L1 retrotransposition assay using the engineered human L1 construct; **B** Retrotransposition assay results for each cell line, up panel: crystal violet stained cells, down panel: statistical analysis for each cell, n = 3 independent experiments. Data are presented as means ± s.d. Statistical analysis was performed by Student’s *t-*test (two-sided), **p < 0.01, *** *p* < 0.001 indicate a significant difference compared to the WT group; **C** RIP-qPCR detecting the binding of YTHDF2 to the L1 retrotransposon using L1 5′ UTR and L1 ORF1p region primers, *NANOG* was used as the positive control. n = 3 independent experiments. Data are presented as means ± s.d. Statistical analysis was performed by Student’s *t* test (two-sided), *p < 0.05, ***p < 0.001 indicating a significant difference compared to the WT group; **D** A genome browser snapshot of YTHDF2 RIP-seq at the L1HS locus, n = 2 independent experiments; **E** Co-detection of YTHDF2, LC3B and L1 ORF1p mRNA by immunofluorescence and RNA-scope at WT day 10 hSSCLCs. Arrows: YTHDF2, LC3B and L1 ORF1p mRNA co-expressed site. Scale bar = 10 μm

To investigate the impact of RNA m^6^A modification on L1 retrotransposition, a cell-based engineered L1-reporter assay [31] was performed. We utilized the pBS-L1PA1-CH-mneo plasmid which contains a neomycin gene located within the 3′UTR and is present in the antisense direction to the SV40 promoter [32]. When the L1 is successfully integrated into the host chromosome, the cells acquire resistance to neomycin (Fig. 4A). The vector was transfected into each cell line on day 6 SSCLCs, and neomycin was added after 48 h and continued for ten days. In METTL3-depleted cells, the number of neomycin-resistant colonies, representing successful L1 retrotransposition, was increased compared to the WT group (Fig. 4B).

The fate of m^6^A-modified mRNAs is determined by a set of readers, including YTH domain-containing proteins, YTHDF1-3, which play a critical role in directing them to cytosolic compartments for translation and decay [9]. While some controversy surrounds this topic [33, 34], it is known that YTHDF2 degrades m^6^A-containing RNAs in mammalian cells and causes TE mRNA decay through phase-partitioning into cytoplasmic processing bodies (P-bodies) [16, 35, 36]. YTHDF2 was found to be upregulated in METTL3 KO cells (Fig. S5A). We utilized RNA immunoprecipitation (RIP) qPCR and YTHDF2 RIP-seq to demonstrate that YTHDF2 binds to L1 mRNAs in an m^6^A-dependent manner (Fig. 4C, D). Additionally, YTHDF2 RIP-seq results unveiled that YTHDF2 binding sites were significantly enriched near the TSS and TES of protein-coding genes, while the binding sites in L1 were mainly enriched in full-length L1s similar to MeRIP-seq (Fig. S5G). Simultaneously, YTHDF2 binding sites were enriched in the m^6^A consensus motif (Fig. S5H). Immunofluorescence results showed that YTHDF2, L1 mRNA, and LC3B were colocalized and concentrated in granules (Fig. 4E, Fig. S5I), demonstrating that L1 mRNA was degraded by autophagy during SSCLCs induction. These results confirm that m^6^A RNA methylation directly affects L1 mRNA abundance by accelerating its degradation through YTHDF2-mediated autophagy.

**Fig. 5:**
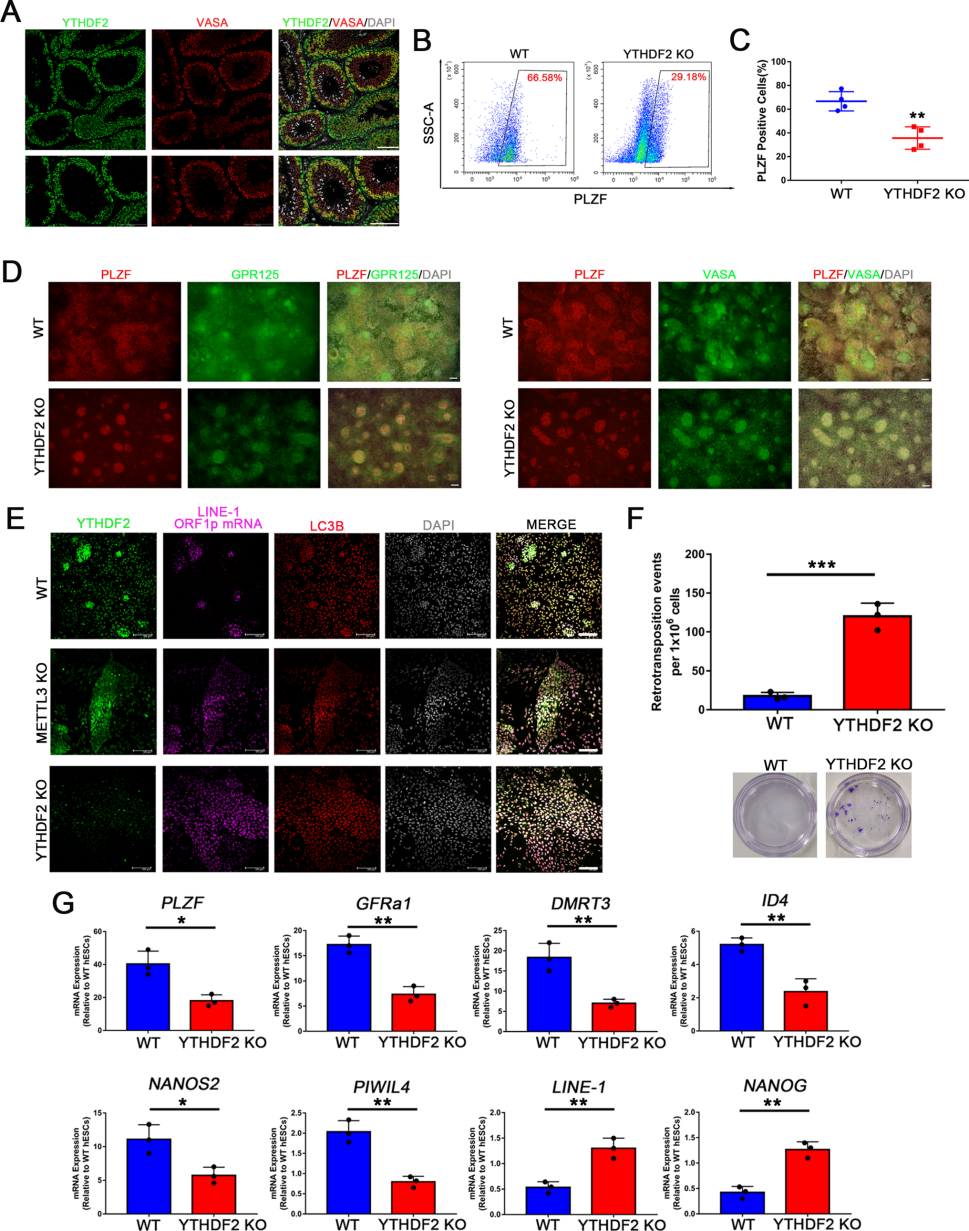
YTHDF2 is essential for SSCLCs differentiation. **A** Immunofluorescence images of adult human testis tubule cross-section stained with YTHDF2 and VASA. Up panel scale bar = 100 μm, down panel scale bar = 50 μm; **B** FACS analysis of WT and YTHDF2 KO hESCs on hSSCLCs induction for 12 days. Boxed areas indicate PLZF positive cells with their percentages; **C** Quantification of FACS results at day 12 of hSSCLCs induction in WT and YTHDF2 KO hESCs; n = 3 independent experiments. Data are presented as means ± s.d. Statistical analysis was performed by Student’s *t-*test (two-sided), **p < 0.01; **D** Immunofluorescence of PLZF, VASA, and GPR125 at day 12 in WT and YTHDF2 KO cells. Scale bar = 100 μm; **E** YTHDF2, LC3B, and L1 ORF1p mRNA co-detected of by immunofluorescence and RNA-scope at day 12 in WT, METTL3 KO, and YTHDF2 KO hSSCLCs. Scale bar = 100 μm; **F** Retrotransposition assay results of WT and YTHDF2 KO groups. Up panel: statistical analysis of each cell, n = 3 independent experiments. Data presented as means ± s.d. Statistical analysis was performed by Student’s *t-*test (two-sided), ***p < 0.001. Down panel: crystal violet stained cells; **G** RT-qPCR analysis of gene expression during hSSCLCs differentiation at day 12; n = 3 independent experiments. Data are presented as means ± s.d. Statistical analysis was performed by Student’s *t-*test (two-sided), *p < 0.05, **p < 0.01, ***p < 0.001

The immunostaining of the human testis revealed that YTHDF2 is localized in the cytoplasm of male germ cells, co-stained with VASA (Fig. 5A). Moreover, the results from 10 × genomics single-cell sequencing indicated that *YTHDF2* exhibits high expression levels in spermatogonial stem cells (marked with *ID4*), spermatogonia (marked with *MAGEA4*), and spermatocytes (marked with *SYCP3*) (Fig. S4E). Consequently, we hypothesized that knocking out YTHDF2, similar to depleting the m^6^A writer METTL3, would increase L1 mRNA abundance. To test this hypothesis, we derived a mutant hES cell line that lacked the YTHDF2 protein (Fig. S5B, C). The depletion of YTHDF2 resulted in loose colonies, similar to METTL3 KO hESCs (Fig. S5D); however, the YTHDF2 KO hESCs still expressed pluripotency markers (Fig. S5E, F). Nevertheless, RNA-seq results demonstrated that METTL3 KO hESCs were more similar to WT than YTHDF2 KO hESCs (Fig. 3A). In YTHDF2 KO cells, up-regulated genes were enriched in RNA splicing and modification, while down-regulated genes were significantly enriched in neuron and cell adhesion regulation (Fig. S6A, B). In contrast, YTHDF2 KO day12 SSCLCs exhibited significant changes in their transcriptomes. A total of 1703 genes were down-regulated, and GO analysis showed items enriched in cell differentiation-related genes, while 1815 up-regulated genes were enriched in RNA processing (Fig. S6C, D).

The depletion of YTHDF2 resulted in a decrease in the induction efficiency of SSCLCs (Fig. 5B, C) and a downregulation of SSC markers, as detected by immunostaining and RT-qPCR in the YTHDF2 KO cell line (Fig. 5D, E, G). The L1-reporter assay showed an increased number of neomycin-resistant colonies in YTHDF2 KO cells compared to the WT group (Fig. 5F). Furthermore, L1 mRNA was highly expressed in day 12 YTHDF2 KO SSCLCs (Fig. 5G). Altogether, these results support the idea that the m^6^A methylation pathway regulates L1 mRNAs in a YTHDF-dependent manner, and YTHDF2 plays a vital role in regulating the expression of L1 mRNAs through the m^6^A methylation pathway.

### ***RNA m***^***6***^***A modification regulates L1 retrotransposons in human germ cells***

**Fig. 6:**
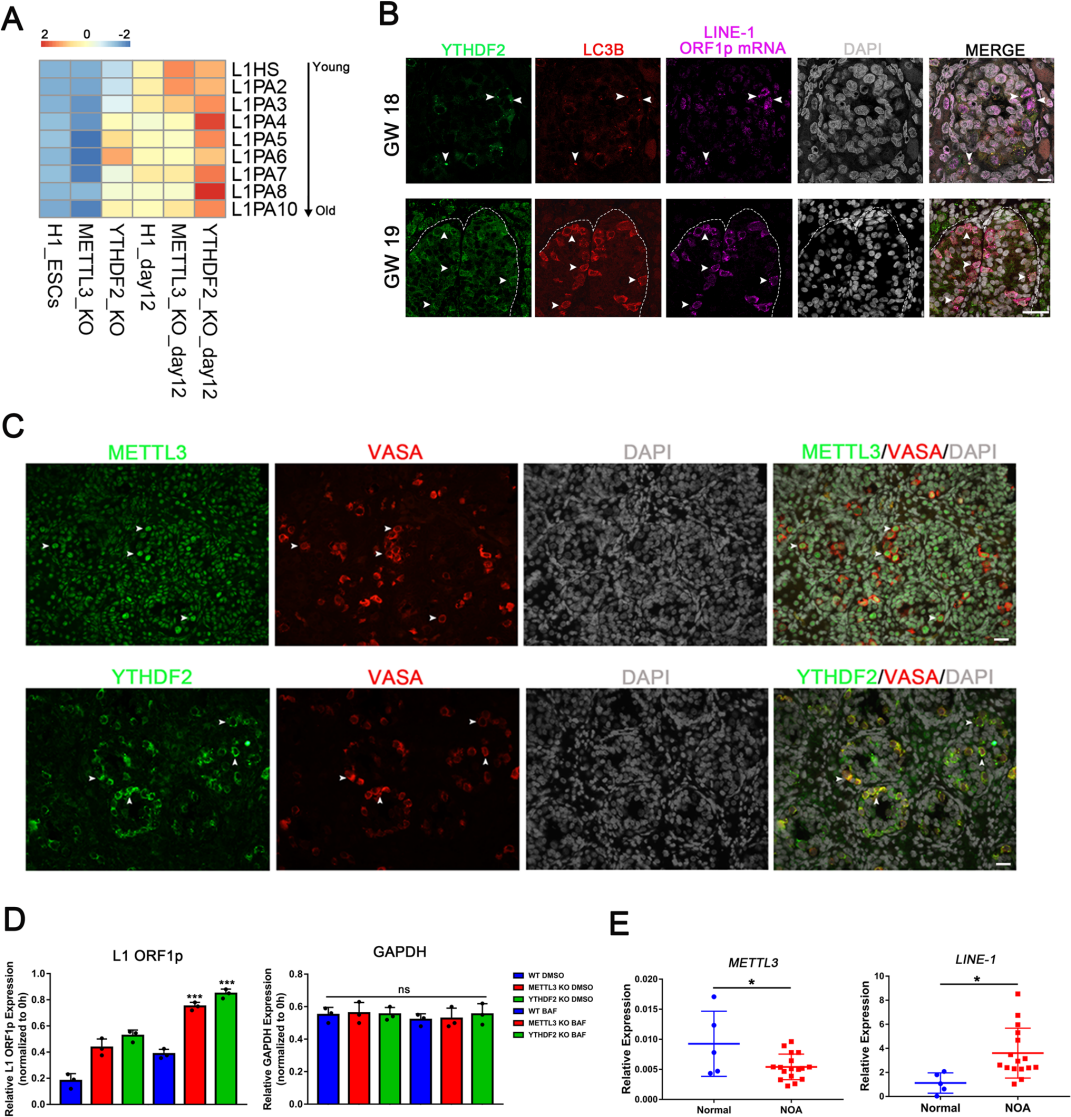
RNA m^6^A modification regulates L1 retrotransposons in human spermatogenesis. **A** A heat map showing the expression levels of different L1 sub-families RNA abundances; **B** Co-detection of YTHDF2, LC3B, and L1 ORF1p mRNA by immunofluorescence and RNA-scope in 18 GW and 19 GW human fetal germ cells. Arrows: YTHDF2, LC3B, and L1 ORF1p mRNA co-expressed cells. Scale bar = 10 μm (up) and 20 μm (down); **C** Immunofluorescence images of fetal human testis tubule cross-section stained with METTL3, YTHDF2, and VASA. Arrows: protein co-expressed cells. Scale bar = 20 μm; **D** RT-qPCR analysis in day 10 SSCLCs treated with dimethylsulphoxide (DMSO) or Bafilomycin (BAF) for one hour and actinomycin D for six hours; n = 3 independent experiments. Data are presented as means ± s.d. Statistical analysis was performed by Student’s *t-*test (two-sided), ***Represent compared to WT BAF group *p* < 0.001; **E** RT-qPCR analysis of METTL3 and L1 expression in meiotic arrest patient’s seminal plasma. Relative expression calculated as 2^^−ΔCT^, ΔCt = Ct (METTL3/L1 – Ct (β-actin). Data presented as means ± s.d. Statistical analysis was performed by Student’s *t-*test (two-sided), *Represent compared to normal group *p* < 0.05

To verify earlier findings and examine whether RNA m^6^A modification regulates L1 retrotransposons in human testis in vivo, we performed immunostaining on human male fetal germ cells in gestational week (GW) of 18–19, which exhibit high levels of L1 transcript [37]. Immunofluorescence revealed that the RNA m^6^A methyltransferase complex proteins METTL3 and WTAP were commonly expressed in the nucleus of fetal testicular tissue. Notably, the fluorescence of METTL3 and WTAP was more intense when co-expressed with VASA (Fig. 6C, Fig. S7A). However, the m^6^A reader YTHDF2 mainly co-localized with VASA and the stress granule marker G3BP1, indicating that YTHDF2 plays a vital role in the development of human fetal germ cells (Fig. 6C, Fig. S7A). By using immunostaining, we discovered that YTHDF2, LC3B, and L1 mRNA were co-localized and concentrated in granules in human fetal germ cells (Fig. 6B, Fig. S7A), which aligns with our in vitro findings. This suggests that RNA m^6^A modification regulates L1 retrotransposons through YTHDF2-dependent autophagy in vivo. Remarkably, RNA-seq results confirmed a significant upregulation of TEs in YTHDF2 knockout hESCs and day12 SSCLCs, with old evolutionary L1s being upregulated (Fig. 6A, Fig. S6E,F). This demonstrates that the YTHDF2 protein is responsible for recognizing and clearance of L1 retrotransposons.

To ensure that the observed increase in L1 RNA levels resulting from the inactivation of METTL3 or YTHDF2 was due to inhibited degradation rather than activated transcription, we conducted an experiment by blocking transcription with actinomycin D and measuring RNA decay. We found that the decay of L1 RNA was suppressed when autophagic degradation was blocked with Bafilomycin (Fig. 6D). This confirmed that the increase in L1 RNA levels was due to inhibited degradation. Additionally, we utilized 2',3'-didehydro-3'-deoxy-thymidine (d4t) to abolish L1 retrotransposition without adversely affecting cell growth and viability. However, the efficiency of SSCLC induction was unaffected by L1 retrotransposition inhibition. This suggests that the primary cause of reduced SSCLC induction efficiency is hESC failure to exit the pluripotent program when the m^6^A methylation pathway is destroyed (Fig. S7C). This conclusion is further supported by the high expression of pluripotent genes in METTL3 KO and YTHDF2 KO SSCLCs (Figs. 3B, 5G, Figs. S3A, C, S6A, C), highlighting the crucial role of m^6^A methylation in regulating cellular differentiation and development.

METTL3 and YTHDF2 were expressed during early human spermatogenesis, and mice knockout experiments showed that the ablation of Mettl3 in germ cells severely inhibited spermatogonial differentiation and blocked the initiation of meiosis [38, 39]. To further investigate whether m^6^A modification regulates L1 retrotransposons in men with meiotic arrest, a subtype of nonobstructive azoospermia, we detected the RNA expression of *METTL3* and L1 in their seminal plasma. Our results showed that L1 retrotransposon RNA was dramatically elevated, while METTL3 was significantly decreased in the seminal plasma of patients with meiotic arrest (Fig. 6E). Whole Exome Sequencing (WES) data from individuals with meiotic arrest identified several potentially pathogenic variants in genes related to m^6^A (Supplementary Table 1). Immunofluorescence of the testis from fertile men demonstrated the expected presence of m^6^A modification, and it was observed that the L1 ORF1p protein was degraded via autophagy (Fig. S7B). Together, these results indicate that RNA m^6^A modification is a crucial mechanism in preventing the mobilization of L1 retrotransposons and maintaining the integrity of the germ cell genome.

## Discussion

METTL3-catalyzed m^6^A is the most prevalent modification on RNA molecules [1]. Although several biological functions of Mettl3-mediated m^6^A methylation have been revealed, the majority of studies on the development of mammalian germ cells have been conducted in mice. This is mainly due to the limited availability and ethical considerations in studying human fetal gonads. Here, using in vitro models and in vivo samples, we presented several findings demonstrating the significant functions of m^6^A during human spermatogenesis and male fertility.

**Fig. 7:**
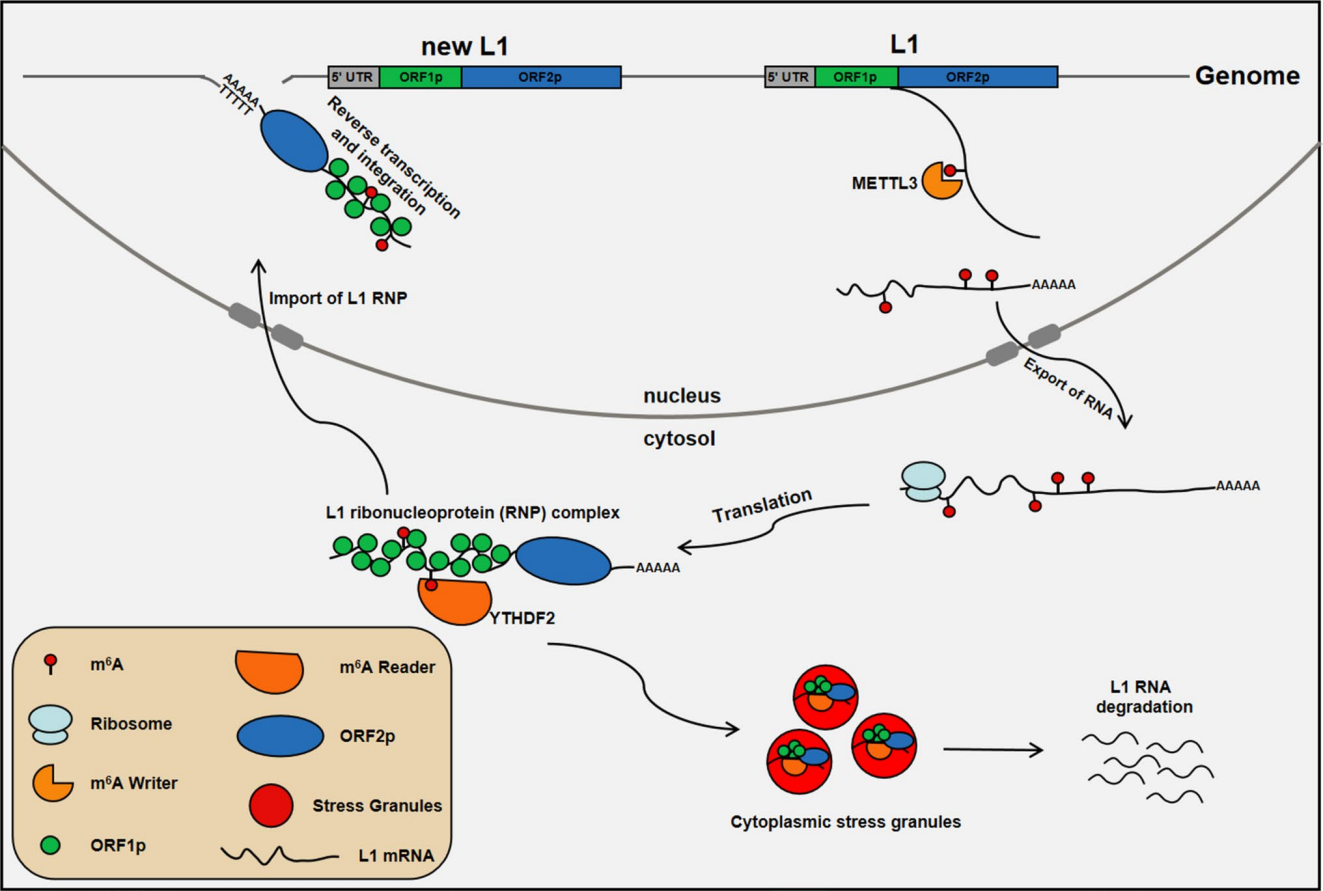
The m^6^A pathway prevents L1 mobilization and subsequent genome disruption. L1 retrotransposons carrying m^6^A modification in necleus by METTL3 and recognized by YTHDF2 in cytosol, subsequently degraded through autophagy

Our results have revealed the essential role of m^6^A RNA modification in the development of early germ cells. Firstly, the deletion of METTL3 in hESCs led to defective differentiation of spermatogonial stem cells in vitro. In addition, we found that METTL3 is highly expressed in the adult human testis and colocalized with VASA, demonstrating its essential role in METTL3-mediated m^6^A modification in male spermatogenesis. Furthermore, transcriptome analysis and MeRIP-seq assays revealed that the deletion of METTL3 resulted in the upregulation of TEs, particularly evolutionarily young L1s, during spermatogonial stem cell differentiation in vitro. Additionally, L1 retrotransposition during hSSCLCs differentiation was degraded via autophagy in an m6A "reader" YTHDF2-dependent manner. We confirmed that L1s containing m6A modification were degraded by YTHDF2-dependent autophagy in human fetal gonads. Meanwhile, men with meiotic arrest showed higher expression of L1 retrotransposon RNA in their seminal plasma. Thus, our study provides evidence that m^6^A is critical in regulating L1 retrotransposon RNA during the early stages of germ cell development and spermatogenesis in humans (Fig. 7).

Previous studies have shown that conditional deletion of Mettl3 and Mettl14 in mouse germ cells severely inhibits spermatogonial differentiation due to dysregulated alternative splicing of genes related to spermatogenesis [38, 39]. Additionally, knockout of Mettl3 and Wtap has been linked to early embryonic lethality in mice [25, 40]. Conditional deletion of m^6^A "reader" proteins, Ythdf2, Ythdc1, and Ythdc2, in mouse germ cells has resulted in aberrant regulation of post-transcriptional m^6^A modification on mRNAs, leading to male infertility [41–44]. Similarly, deleting Alkbh5, one of the m^6^A demethylases, in mouse germ cells also causes infertility due to impaired RNA splicing and apoptosis of spermatocytes [6, 45]. The essential roles of the m^6^A demethylase, methyltransferase, and m^6^A RNA recognition in spermatogenesis suggest that m^6^A modification in RNA is critical for the maintenance of the differentiation program during spermatogenesis.

Even though studies on mESCs have shown that m^6^A modification decreases the stability of L1 RNA concerning R-loop or chromatin regulation [46–48], research regarding the role of m^6^A modification on TEs in mammalian germ cells is still limited. Recent studies have revealed that depleting m^6^A methyltransferases METTL3 and METTL14 leads to increased mRNA abundance of IAPs and ERVK elements by targeting their 5′ untranslated regions, reducing the half-life of IAP mRNA, and this occurs by the recruitment of the YTHDF family of m^6^A reader proteins [16]. Meanwhile, the m^6^A demethylase FTO regulates L1 RNA abundance in mESCs by mediating m^6^A demethylation of L1 RNA and also plays regulatory roles in shaping chromatin state and gene expression [17]. Two recent studies have found that the nuclear m^6^A reader YTHDC1 binds to retrotransposon transcripts, such as IAP, ERVK, and L1, in mESCs. Depletion of YTHDC1 results in the reactivation of silenced retrotransposons [18, 49]. Intriguingly, YTHDC1 binds the m^6^A-marked L1 RNAs on chromatin, thereby regulating the formation of the L1-NCL-KAP1/SETDB1 complex to establish H3K9me3 modifications on two-cell stage (2C) related retrotransposons. This represses the 2C program and ensures the appropriate transcriptome and developmental potency of ESCs and early embryos [18]. In contrast, studies on human cancer cells have found that L1 retrotransposons hijack the RNA m^6^A modification system for successful replication, facilitating retrotransposition and contributing to long gene vulnerability [19, 20]. Therefore, the m^6^A modification on L1 retrotransposons has different functions in various human cells and distinct stages of development.

As parasitic genetic elements, TEs such as L1s pose a threat to the stability of the host genome, particularly during spermatogenesis. Hosts have developed sophisticated strategies, including DNA methylation and demethylation, histone modification, and the PIWI-piRNA pathway, along with other transcriptional factors, to suppress their expression [13, 50]. In this paradigm, L1s are viewed as a threat to developing germ cells and must be silenced immediately by the m^6^A pathway to prevent their mobilization and subsequent genome disruption [51]. Thus, our findings demonstrate a novel mechanism for METTL3 in the early stages of germ cell development and spermatogenesis in humans by modulating L1 retrotransposons. Specifically, we have discovered that L1 retrotransposons with m^6^A modification can be recognized and degraded through autophagy during hSSCLCs induction and in human fetal gonads via YTHDF2. Moreover, the knockout of YTHDF2 increased both evolutionarily young and old L1 retrotranspositions and decreased the efficiency of SSC induction. By analyzing seminal plasma and WES data, we revealed that the loss of m^6^A modification reactivated L1 retrotransposons, suggesting that defective spermatogenesis may be a result in patients with meiotic arrest. Our findings provide novel insights into the role and regulatory mechanisms of m^6^A modification in L1 retrotransposition restriction, with m^6^A modification acting as a safeguard for genome stability during human germline development. Thus, our results provide strong evidence for deciphering the in vitro and in vivo functions of METTL3-mediated m^6^A modification in human spermatogenesis.

### Supplementary Information

Below is the link to the electronic supplementary material.

Supplementary file1 (TIF 5987 KB) Figure S1 Generation of METTL3 Gene Knockout hES Cell Line. (A) Right: The DNA sequences of both alleles for the indicated knockout lines. Red letters indicate the positions of the guide RNAs. del: deletion; ins: insertion; Left: Sanger sequencing of KO hESCs in METTL3 gene target locus. Arrow indicates the delete site, box indicates the insert site; (B) Western blots showing the expression level of METTL3 in each hESC line; (C) m6A dot blot shows the mRNA m6A levels in each cell line, MB: methylene blue; (D) ELISA shows the normalized m6A levels in mRNA in each hESC line. Data presented as means ± s.d. Statistical analysis was performed by Student’s t-test (two-sided), *** represent compared to WT group p < 0.001; (E) Immunofluorescence of SOX2, NANOG, POU5F1, and SSEA4 in METTL3 KO hESCs. Scale bar=50 μm; (F) Vector for the overexpression of wild-type METTL3 and mutant METTL3.

Supplementary file2 (TIF 487 KB) Figure S2 METTL3 is indispensable for hPGCLC induction. (A) FACS analysis of WT and METTL3 KO hESCs on hPGCLCs induction for 4 days. Boxed areas indicate EpCAM/INTEGRINα (+) cells with their percentages; (B) Quantification of FACS at day 4 of hPGCLC induction in WT and METTL3 KO hESCs; n = 3 independent experiments. Data are presented as means ± s.d; (C) RT-qPCR analysis of gene expression during hPGC differentiation on day 4 embryoid; n = 3 independent experiments. Data presented as means ± s.d.

Supplementary file3 (TIF 1202 KB) Figure S3 RNA-seq and MeRIP-seq results in METTL3 KO hESCs. (A) Volcano plot of the expression of genes upregulated (red) or downregulated (blue) (fold changes > 2) in METTL3 KO hESCs compared to WT hESCs; (B) Differentially expressed gene levels in METTL3 KO hESCs compared to WT hESCs is represented by a heat map. The GO (Gene Ontology) functional terms and representative genes are shown for each gene cluster; (C) Volcano plot of the expression of genes upregulated (red) or downregulated (blue) (fold changes > 2) in METTL3 KO d12 compared to WT d12; (D) A substantially higher increase in the expression of m6A-mRNAs upon METTL3 KO. The expression distribution of each m6A-mRNA subgroup is quantified in the boxplots. n = 2 independent experiments. Data presented as means ± s.d. Statistical analysis was performed by Student’s t-test (two-sided), *** p < 0.001, represent compared to WT group; (E) The predominant consensus motif DRACH detected by HOMER in MeRIP-seq.

Supplementary file4 (TIF 5588 KB) Figure S4 L1 retrotransposon was degraded by autophagy during hSSCLC induction. (A) m6A RIP-qPCR detecting the binding of m6A methylation to the L1 retrotransposon using L1 5′ UTR and L1 ORF1p region primers, EEF1A1 as the positive control. n = 3 independent experiments. Data are presented as means ± s.d. Statistical analysis was performed by Student’s t test (two-sided), *p < 0.05, *** p < 0.001 indicating a significant difference compared to the WT group; (B) Immunofluorescence of PLZF and ORF1p at day 12 for WT and METTL3 KO cells. Scale bar=20 μm; (C) Western blot showing autophagy marker LC3B, p62, L1 ORF1p, and PLZF expression level in each cell line; (D) Volcano plot showing log2FC in transposable elements expression in METTL3 KO hESCs or day 12 SSCLCs versus WT hESCs or day 12 SSCLCs using a random assignment of multi-mapped reads; (E) Dimension reduction representation of single-cell RNA-seq (t-SNE, t-Distributed Stochastic Neighbor Embedding) measured in the adult human testis, showing the co-expression of the m6A “reader” YTHDF2 in germ cells. ID4 represents spermatogonial stem cells, MAGEA4 represents spermatogonia, and SYCP3 represents spermatocytes.

Supplementary file5 (TIF 4514 KB) Figure S5 Generation of YTHDF2 Gene Knockout hES Cell Line. (A) The expression levels of YTHDF1, YTHDF2, and YTHDF3 in each cell line were determined by RNA-seq. n = 2 independent experiments. Data are presented as means ± s.d. Statistical analysis was performed by Student’s t-test (two-sided), **p < 0.01, *** p < 0.001 indicating a significant difference compared to the WT group; (B) Design of the CRISPR target for the YTHDF2 gene, red arrows indicate the sgRNA locus; (C) Western blot showing the expression level of YTHDF2 in WT and YTHDF2 KO hESCs; (D) Bright field images of WT and YTHDF2 KO hESCs, Scale bar=100 μm; (E) FACS analysis for the expression of POU5F1, SOX2, NANOG, CD24, and CD90 in YTHDF2 KO hESCs; (F) Immunofluorescence images of SOX2, NANOG, and POU5F1 in YTHDF2 KO hESCs. Scale bar=50 μm; (G) Normalized YTHDF2 RIP-seq read count in each cell line across the -3 kb upstream of the transcription start sites (TSS), through scaled gene bodies (5 kb) to +3 kb downstream of transcription end sites (TES) of mRNA and the 5′ UTR, ORF1, ORF2, and 3′ UTR of L1 mRNA with at least one peak; (H) The predominant consensus motif DRACH detected by HOMER in YTHDF2 RIP-seq; (I) RNAscope positive and negative control results, Scale bar=10 μm.

Supplementary file6 (TIF 2534 KB) Figure S6 The RNA-seq results in YTHDF2 KO hESCs. (A) Volcano plot showing the expression of genes upregulated (red) or downregulated (blue) (fold changes > 2) in YTHDF2 KO hESCs compared to WT hESCs; (B) GO functional terms of differentially expressed genes; (C) Volcano plot showing the expression of genes upregulated (red) or downregulated (blue) (fold changes > 2) in YTHDF2 KO day 12 compared to WT day 12; (D) GO functional terms of differentially expressed genes; (E) Volcano plot showing log2FC in transposable elements expression in YTHDF2 KO hESCs versus WT hESCs using a random assignment of multi-mapped reads; (F) Volcano plot showing log2FC in transposable elements expression in YTHDF2 KO day 12 versus WT day 12 SSCLCs using a random assignment of multi-mapped reads.

Supplementary file7 (TIF 6760 KB) Figure S7 (A) Immunofluorescence images of fetal human testis tubule cross-section stained with WTAP, VASA, YTHDF2, G3BP1, and LC3B. Arrows: protein co-expressed sites. Scale bar=20 μm;; (B) Immunofluorescence images of fertile man testis tubule cross-section stained with m6A, VASA, ORF1p, and LC3B. Arrows: protein co-expressed sites. Scale bar=50 μm; (C) Right: FACS analysis of day 12 SSCLCs in each cell line by adding 2',3'-didehydro-3'-deoxy-thymidine (d4t) to abolish retrotransposition of L1; Left: Quantification of FACS at day 12 of SSCLC induction in WT, METTL3 KO, and YTHDF2 KO hESCs with or without d4t; n = 3 independent experiments. Data are presented as means ± s.d. Statistical analysis was performed by Student’s t-test (two-sided), ns represents no significant difference compared to the -d4t group.

Supplementary file8 (TIF 1806 KB) Figure S8 Original images of Western Blotting

Supplementary file9 (DOCX 26 KB)

Supplementary file10 (CSV 1112 KB)

## Data Availability

The China National Center for Bioinformation accession number for the sequencing data reported in this paper is HRA002601.

## Abbreviations

METTL3: Methyltransferases like 3
LINE-1, L1: Long interspersed element type 1
m^6^A: N6-methyl-adenosine
TEs: Transposable elements
hESCs: Human embryonic stem cells
hSSCs: Human spermatogonial stem cells
DSBs: DNA double-strand breaks
ERVs: Endogenous retroviruses
IAPs: Intracisternal A-particles
WES: Whole exome sequencing
ELISA: Enzyme-linked immunosorbent assay
hPGCs: Human primordial germ cells
hSSCLCs: Human spermatogonial stem cell-like cells
PLZF: Promyelocytic leukemia zinc finger
PCA: Principal component analysis
GO: Gene ontology
MeRIP: Methylated RNA immunoprecipitation
