# Supplementary figures and images for: RNA m6A modification regulates L1 retrotransposons in human spermatogonial stem cell differentiation in vitro and in vivo
Source: Cell Mol Life Sci. 2024 Feb 16;81(1):92. doi: 10.1007/s00018-024-05119-0 (PMC10873452; doi:10.1007/s00018-024-05119-0)

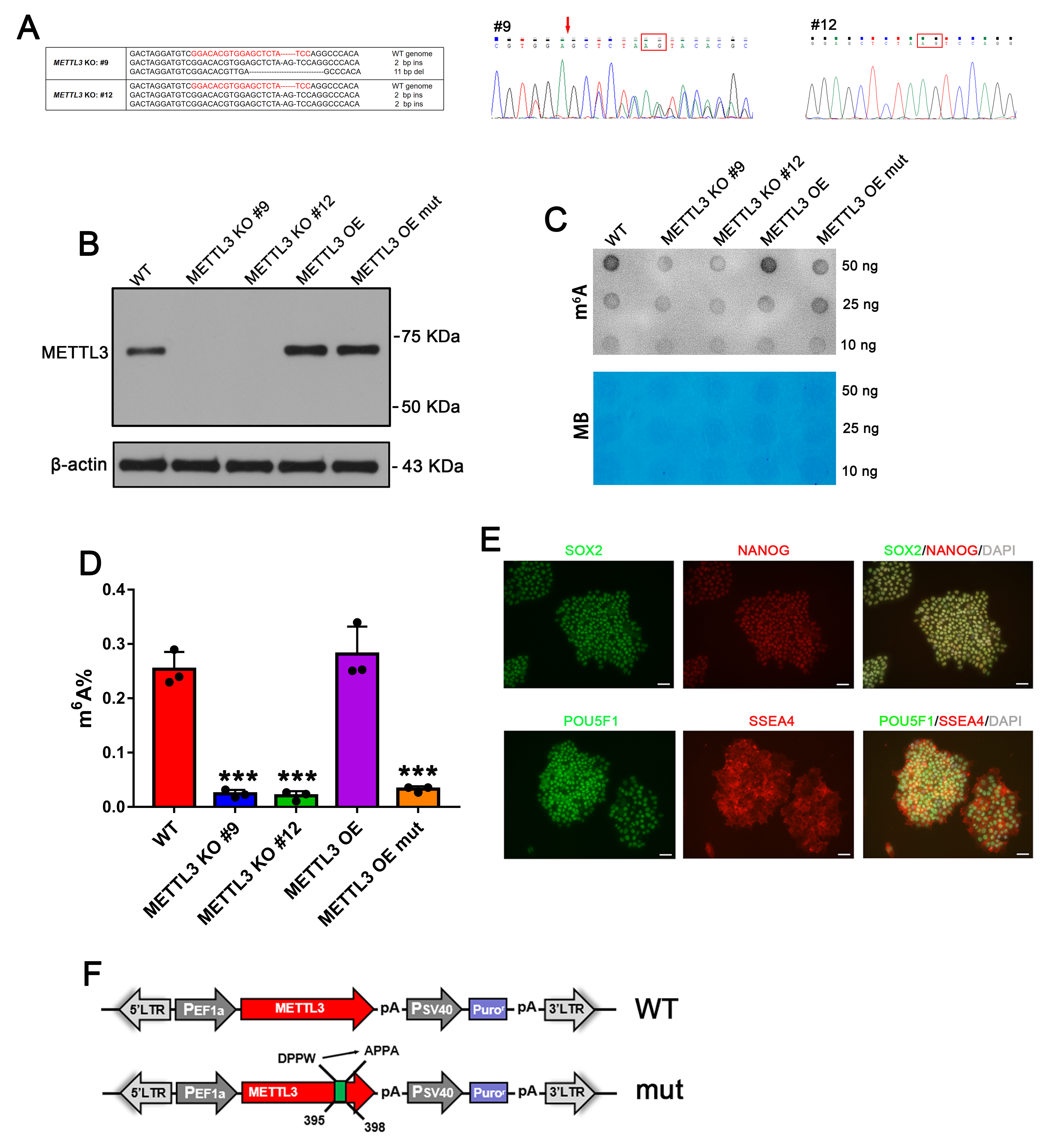

Supplement: Supplementary file 1 — Supplementary file1 (TIF 5987 KB) Figure S1 Generation of METTL3 Gene Knockout hES Cell Line. (A) Right: The DNA sequences of both alleles for the indicated knockout lines. Red letters indicate the positions of the guide RNAs. del: deletion; ins: insertion; Left: Sanger sequencing of KO hESCs in METTL3 gene target locus. Arrow indicates the delete site, box indicates the insert site; (B) Western blots showing the expression level of METTL3 in each hESC line; (C) m6A dot blot shows the mRNA m6A levels in each cell line, MB: methylene blue; (D) ELISA shows the normalized m6A levels in mRNA in each hESC line. Data presented as means ± s.d. Statistical analysis was performed by Student’s t-test (two-sided), *** represent compared to WT group p < 0.001; (E) Immunofluorescence of SOX2, NANOG, POU5F1, and SSEA4 in METTL3 KO hESCs. Scale bar=50 μm; (F) Vector for the overexpression of wild-type METTL3 and mutant METTL3. [file 18_2024_5119_MOESM1_ESM.tif]

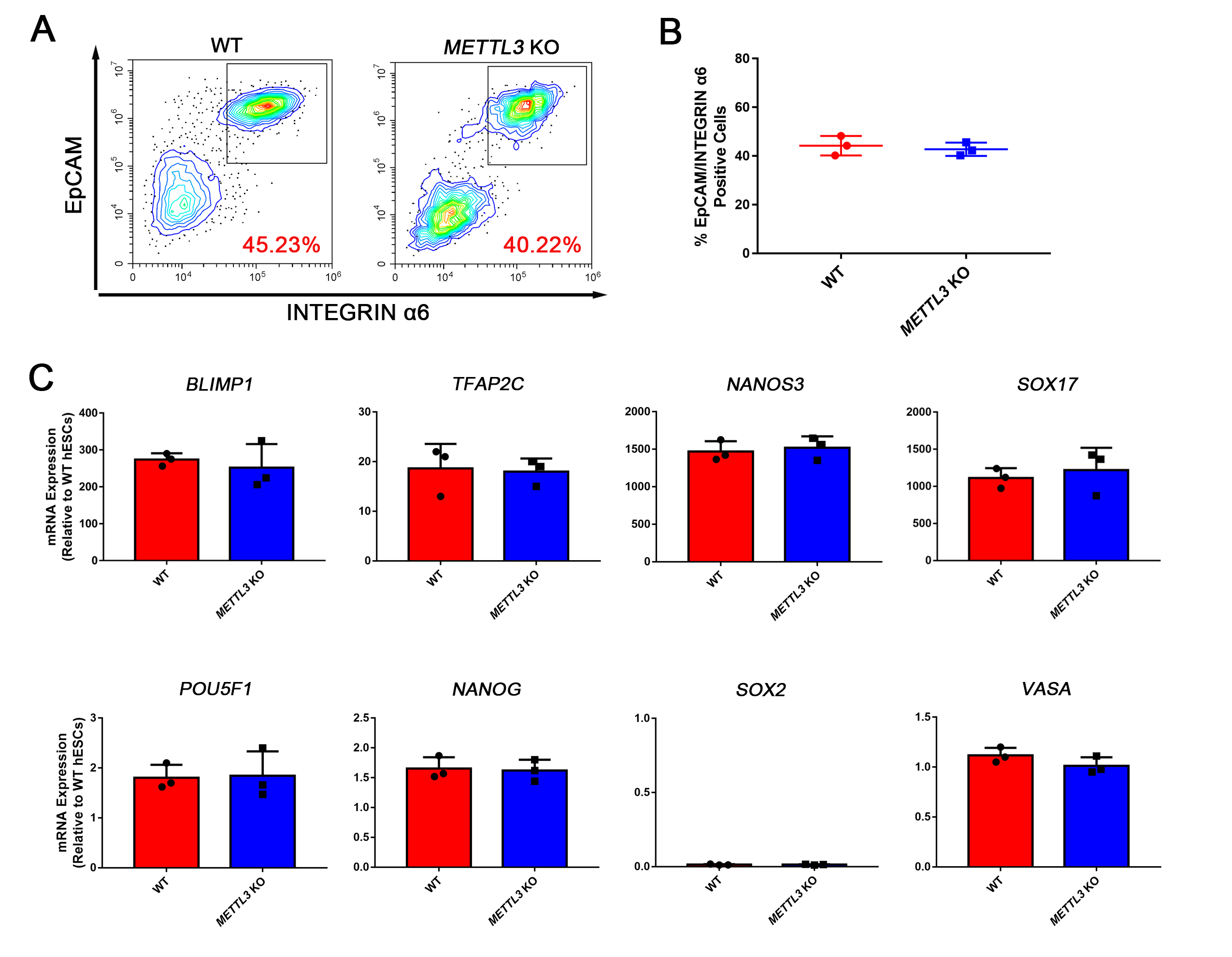

Supplement: Supplementary file 2 — Supplementary file2 (TIF 487 KB) Figure S2 METTL3 is indispensable for hPGCLC induction. (A) FACS analysis of WT and METTL3 KO hESCs on hPGCLCs induction for 4 days. Boxed areas indicate EpCAM/INTEGRINα (+) cells with their percentages; (B) Quantification of FACS at day 4 of hPGCLC induction in WT and METTL3 KO hESCs; n = 3 independent experiments. Data are presented as means ± s.d; (C) RT-qPCR analysis of gene expression during hPGC differentiation on day 4 embryoid; n = 3 independent experiments. Data presented as means ± s.d. [file 18_2024_5119_MOESM2_ESM.tif]

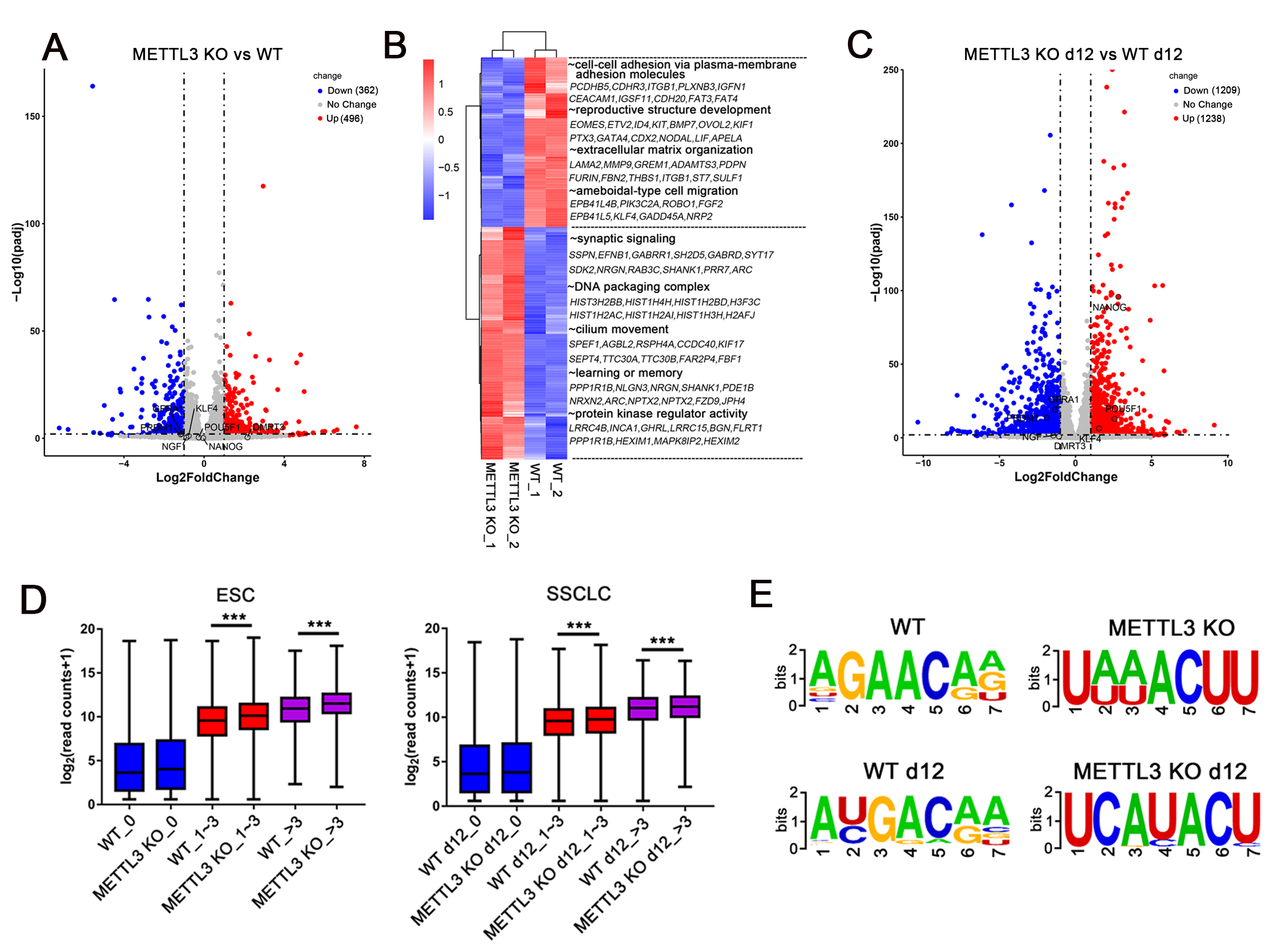

Supplement: Supplementary file 3 — Supplementary file3 (TIF 1202 KB) Figure S3 RNA-seq and MeRIP-seq results in METTL3 KO hESCs. (A) Volcano plot of the expression of genes upregulated (red) or downregulated (blue) (fold changes > 2) in METTL3 KO hESCs compared to WT hESCs; (B) Differentially expressed gene levels in METTL3 KO hESCs compared to WT hESCs is represented by a heat map. The GO (Gene Ontology) functional terms and representative genes are shown for each gene cluster; (C) Volcano plot of the expression of genes upregulated (red) or downregulated (blue) (fold changes > 2) in METTL3 KO d12 compared to WT d12; (D) A substantially higher increase in the expression of m6A-mRNAs upon METTL3 KO. The expression distribution of each m6A-mRNA subgroup is quantified in the boxplots. n = 2 independent experiments. Data presented as means ± s.d. Statistical analysis was performed by Student’s t-test (two-sided), *** p < 0.001, represent compared to WT group; (E) The predominant consensus motif DRACH detected by HOMER in MeRIP-seq. [file 18_2024_5119_MOESM3_ESM.tif]

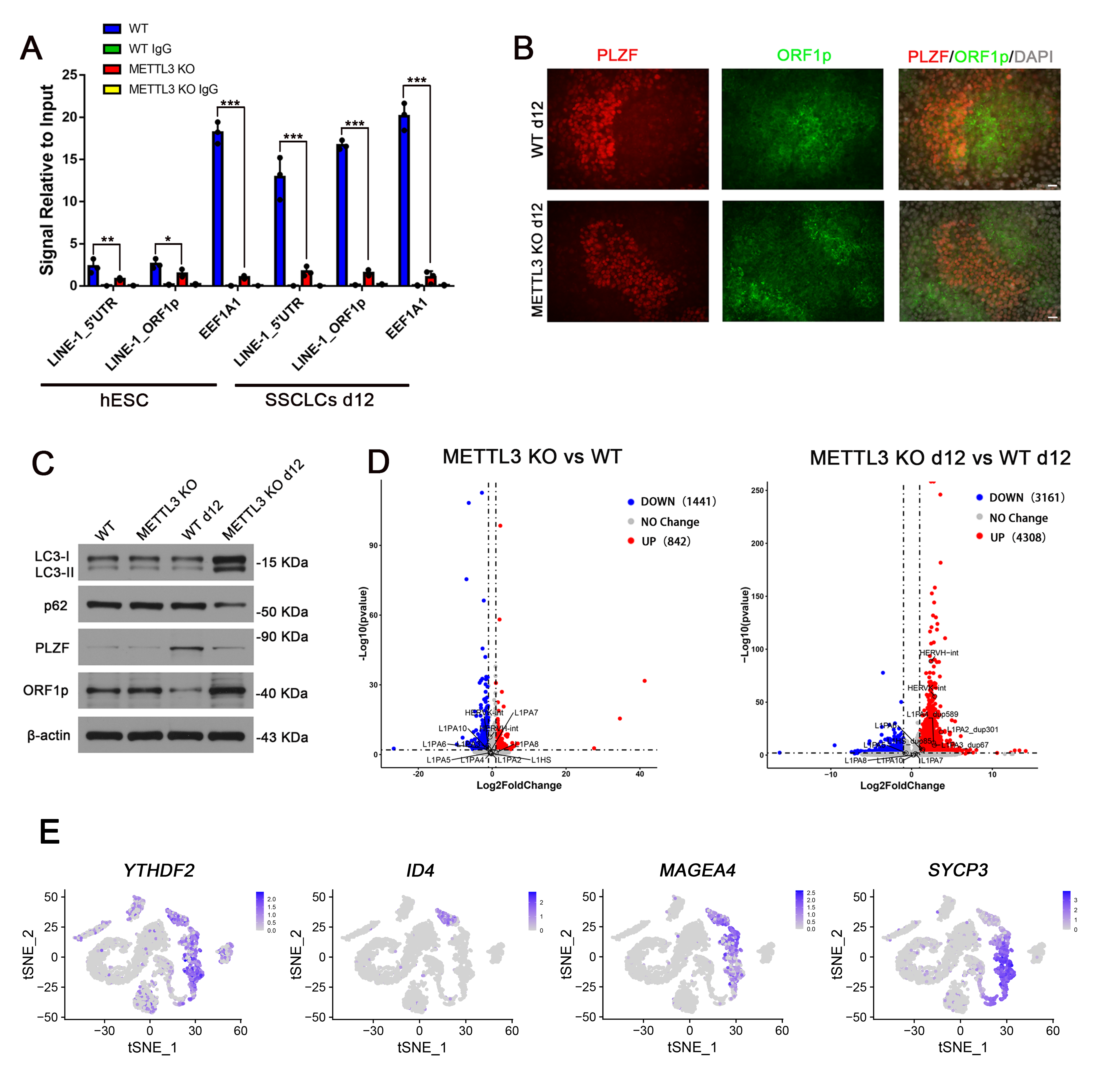

Supplement: Supplementary file 4 — Supplementary file4 (TIF 5588 KB) Figure S4 L1 retrotransposon was degraded by autophagy during hSSCLC induction. (A) m6A RIP-qPCR detecting the binding of m6A methylation to the L1 retrotransposon using L1 5′ UTR and L1 ORF1p region primers, EEF1A1 as the positive control. n = 3 independent experiments. Data are presented as means ± s.d. Statistical analysis was performed by Student’s t test (two-sided), *p < 0.05, *** p < 0.001 indicating a significant difference compared to the WT group; (B) Immunofluorescence of PLZF and ORF1p at day 12 for WT and METTL3 KO cells. Scale bar=20 μm; (C) Western blot showing autophagy marker LC3B, p62, L1 ORF1p, and PLZF expression level in each cell line; (D) Volcano plot showing log2FC in transposable elements expression in METTL3 KO hESCs or day 12 SSCLCs versus WT hESCs or day 12 SSCLCs using a random assignment of multi-mapped reads; (E) Dimension reduction representation of single-cell RNA-seq (t-SNE, t-Distributed Stochastic Neighbor Embedding) measured in the adult human testis, showing the co-expression of the m6A “reader” YTHDF2 in germ cells. ID4 represents spermatogonial stem cells, MAGEA4 represents spermatogonia, and SYCP3 represents spermatocytes. [file 18_2024_5119_MOESM4_ESM.tif]

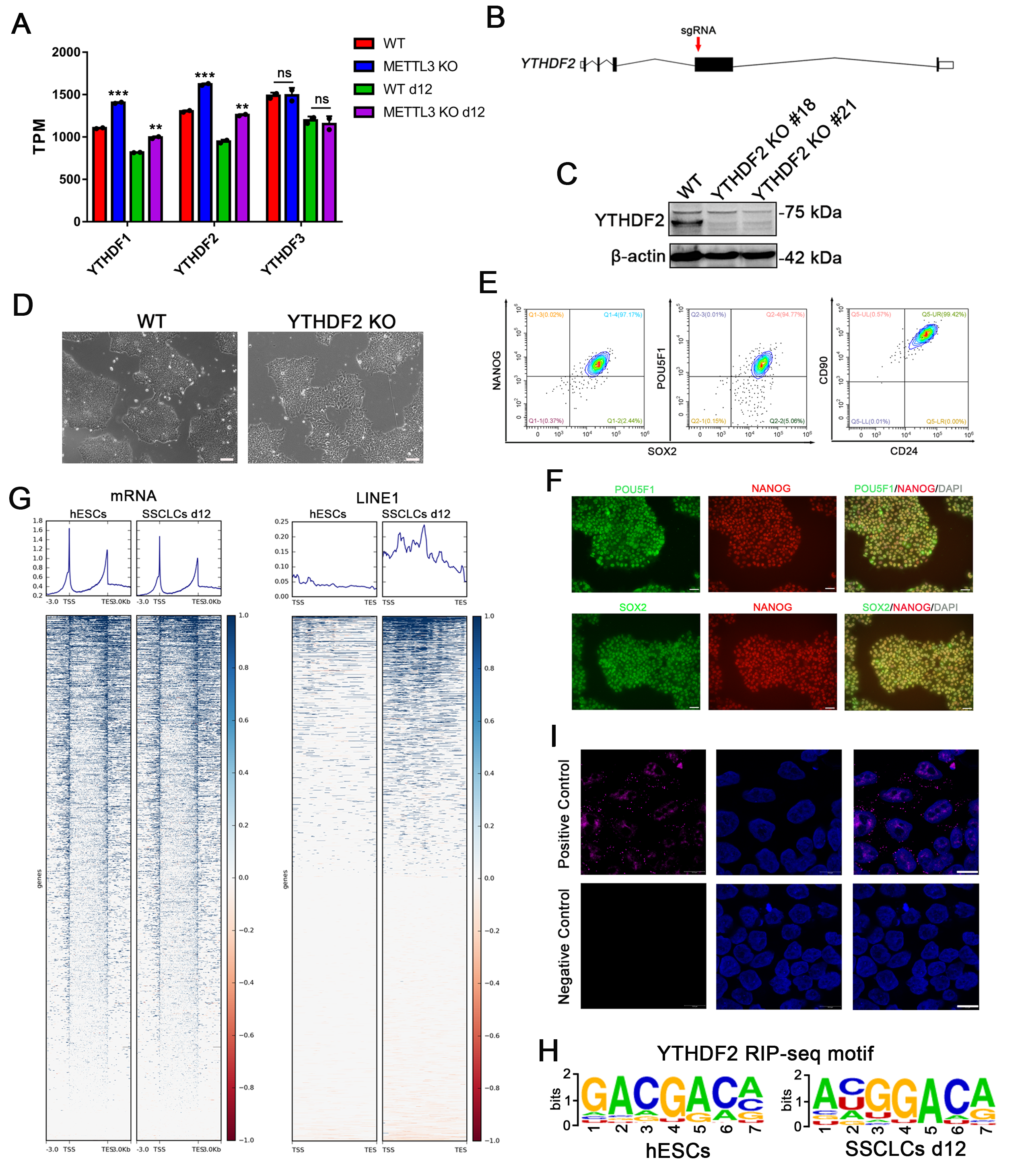

Supplement: Supplementary file 5 — Supplementary file5 (TIF 4514 KB) Figure S5 Generation of YTHDF2 Gene Knockout hES Cell Line. (A) The expression levels of YTHDF1, YTHDF2, and YTHDF3 in each cell line were determined by RNA-seq. n = 2 independent experiments. Data are presented as means ± s.d. Statistical analysis was performed by Student’s t-test (two-sided), **p < 0.01, *** p < 0.001 indicating a significant difference compared to the WT group; (B) Design of the CRISPR target for the YTHDF2 gene, red arrows indicate the sgRNA locus; (C) Western blot showing the expression level of YTHDF2 in WT and YTHDF2 KO hESCs; (D) Bright field images of WT and YTHDF2 KO hESCs, Scale bar=100 μm; (E) FACS analysis for the expression of POU5F1, SOX2, NANOG, CD24, and CD90 in YTHDF2 KO hESCs; (F) Immunofluorescence images of SOX2, NANOG, and POU5F1 in YTHDF2 KO hESCs. Scale bar=50 μm; (G) Normalized YTHDF2 RIP-seq read count in each cell line across the -3 kb upstream of the transcription start sites (TSS), through scaled gene bodies (5 kb) to +3 kb downstream of transcription end sites (TES) of mRNA and the 5′ UTR, ORF1, ORF2, and 3′ UTR of L1 mRNA with at least one peak; (H) The predominant consensus motif DRACH detected by HOMER in YTHDF2 RIP-seq; (I) RNAscope positive and negative control results, Scale bar=10 μm. [file 18_2024_5119_MOESM5_ESM.tif]

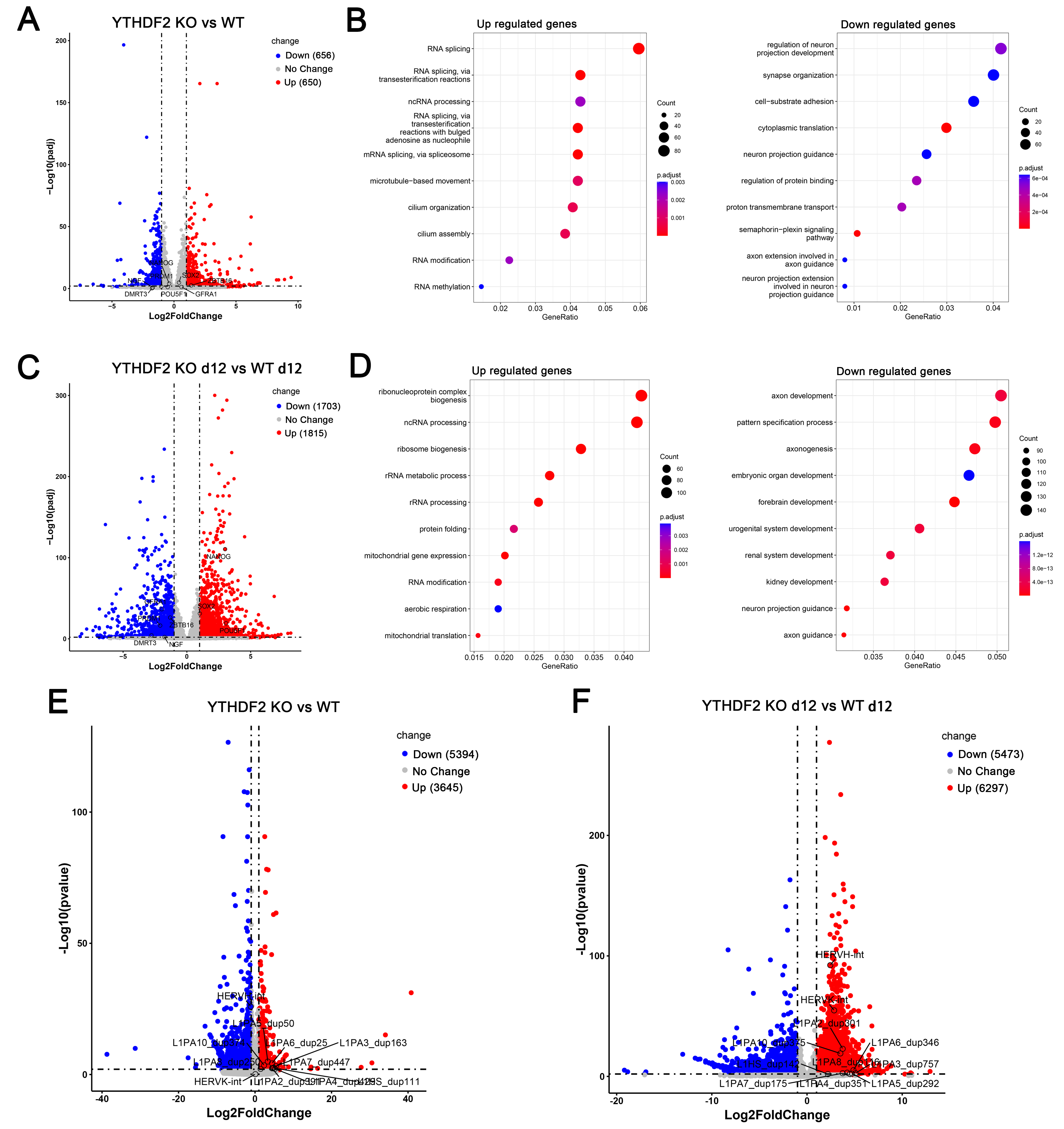

Supplement: Supplementary file 6 — Supplementary file6 (TIF 2534 KB) Figure S6 The RNA-seq results in YTHDF2 KO hESCs. (A) Volcano plot showing the expression of genes upregulated (red) or downregulated (blue) (fold changes > 2) in YTHDF2 KO hESCs compared to WT hESCs; (B) GO functional terms of differentially expressed genes; (C) Volcano plot showing the expression of genes upregulated (red) or downregulated (blue) (fold changes > 2) in YTHDF2 KO day 12 compared to WT day 12; (D) GO functional terms of differentially expressed genes; (E) Volcano plot showing log2FC in transposable elements expression in YTHDF2 KO hESCs versus WT hESCs using a random assignment of multi-mapped reads; (F) Volcano plot showing log2FC in transposable elements expression in YTHDF2 KO day 12 versus WT day 12 SSCLCs using a random assignment of multi-mapped reads. [file 18_2024_5119_MOESM6_ESM.tif]

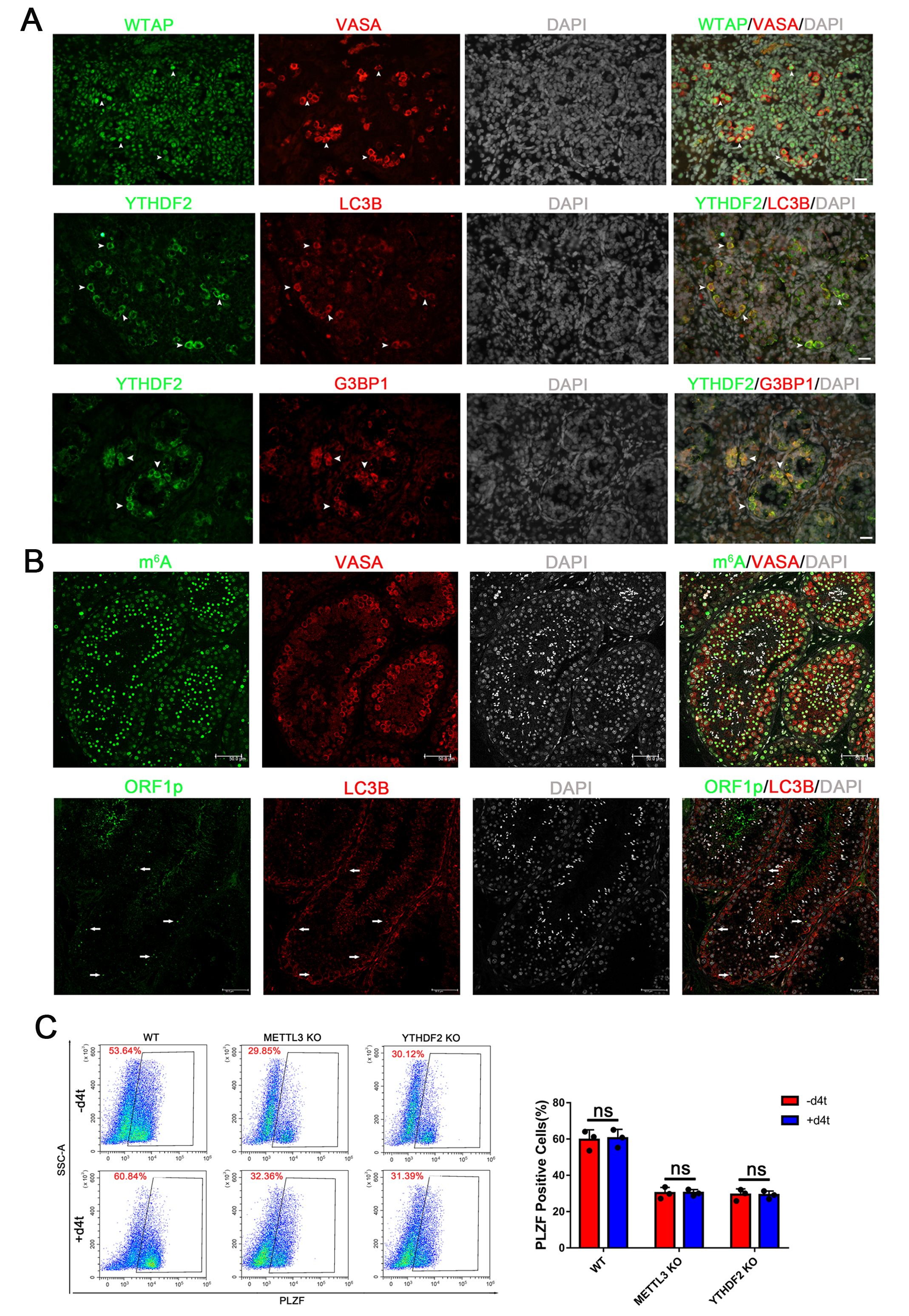

Supplement: Supplementary file 7 — Supplementary file7 (TIF 6760 KB) Figure S7 (A) Immunofluorescence images of fetal human testis tubule cross-section stained with WTAP, VASA, YTHDF2, G3BP1, and LC3B. Arrows: protein co-expressed sites. Scale bar=20 μm;; (B) Immunofluorescence images of fertile man testis tubule cross-section stained with m6A, VASA, ORF1p, and LC3B. Arrows: protein co-expressed sites. Scale bar=50 μm; (C) Right: FACS analysis of day 12 SSCLCs in each cell line by adding 2',3'-didehydro-3'-deoxy-thymidine (d4t) to abolish retrotransposition of L1; Left: Quantification of FACS at day 12 of SSCLC induction in WT, METTL3 KO, and YTHDF2 KO hESCs with or without d4t; n = 3 independent experiments. Data are presented as means ± s.d. Statistical analysis was performed by Student’s t-test (two-sided), ns represents no significant difference compared to the -d4t group. [file 18_2024_5119_MOESM7_ESM.tif]

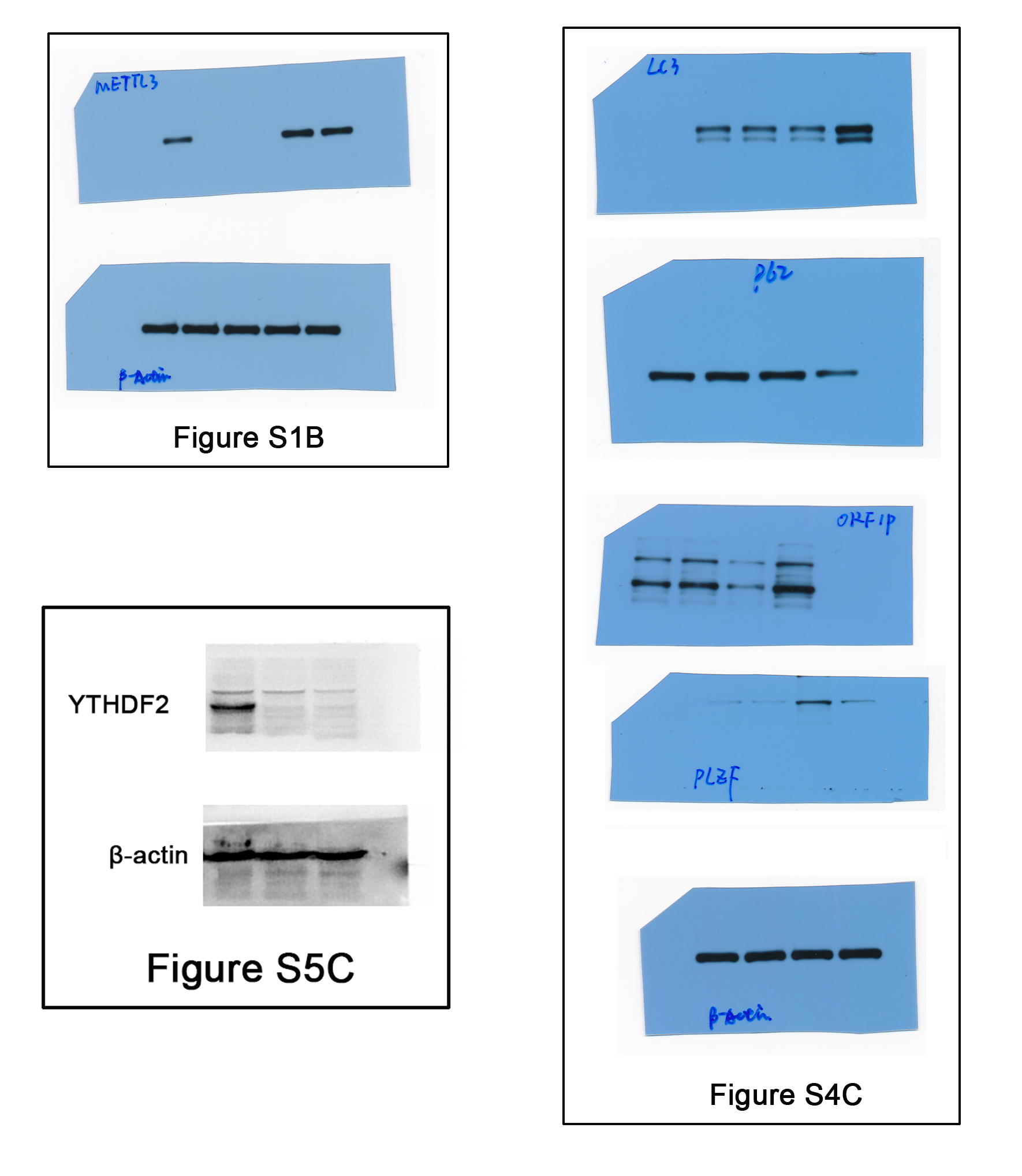

Supplement: Supplementary file 8 — Supplementary file8 (TIF 1806 KB) Figure S8 Original images of Western Blotting [file 18_2024_5119_MOESM8_ESM.tif]
