# Supplementary material for: RNA m6A modification regulates L1 retrotransposons in human spermatogonial stem cell differentiation in vitro and in vivo
Source: Cell Mol Life Sci. 2024 Feb 16;81(1):92. doi: 10.1007/s00018-024-05119-0 (PMC10873452; doi:10.1007/s00018-024-05119-0)
Supplement: Supplementary file 9 — Supplementary file9 (DOCX 26 KB) [file 18_2024_5119_MOESM9_ESM.docx]

**Supplementary Tables**

Supplementary Table1：Identified variants in m^6^A related genes associated with meiotic arrest patients

| **Patient** | **Gene** | **Change in DNA** | **AA Alteration** | **InterVar** | **SNP** | **SIFT** | **CADD** | **PolyPhen-2** | **MutationTaster** |
| --- | --- | --- | --- | --- | --- | --- | --- | --- | --- |
| Patient20 | YTHDF1 | chr20:61834673:C>T | p.V207I | Uncertain significance | rs187686193 | 0.282 | 12.06 | B | D |
|  | ALKBH5 | chr17:18088094:C/T | p.C537T | Benign | rs11078411 | - | - | - | - |
|  | PBM15 | chr1:110882884:G>A | p.G286D | Uncertain significance | - | 0.27 | 4.475 | B | N |
| Patient21 | YTHDC2 | chr5:112917114:G>A | p.A819T | Uncertain significance | - | 0.608 | 16.24 | B | D |
|  | WTAP | chr6:160176277:C>T | p.C825T | Likely benign | rs138053621 | - | - | - | - |
|  | FTO | chr16:53860211:G>A | p.G187R | Uncertain significance | rs577569584 | 0.467 | 9.844 | B | B |
| Patient22 | YTHDC2 | chr5:112889696:A>G | p.I404V | Uncertain significance | - | 0.105 | 23.5 | P | D |
|  | VIRMA | chr8:95518906:T>C | p.I1307V | Likely benign | rs145312139 | 1 | 0.001 | B | N |
| Patient23 | FTO | chr16:53860082:G>A | p.D144N | Uncertain significance | rs201086068 | 0.069 | 23.8 | P | D |
| Patient25 | WTAP | chr6:160176277:C>T | p.C825T | Likely benign | rs138053621 | - | - | - | - |
| Patient26 | YTHDF2 | chr1:29069127:A>G | p.A195G | Likely benign | rs200930096 | - | - | - | - |
|  | YTHDF2 | chr1:29069965:A>T | p.I395F | Uncertain significance | rs563856287 | 0.058 | 17.27 | B | D |
| Patient27 | VIRMA | chr8:95523504:G>A | p.S1100F | Uncertain significance | rs777594318 | 0.013 | 25.2 | B | D |

Note: SIFT score ranges from 0.00 to 1.00. Amino acid substitution is predicted to be damaging if the score is <0.05 and tolerated if the score is >0.05. CADD score: amino acid substitution is predicted to be damaging if the score is >15. Polyphen-2 score ranges from 0.000 to 1.000: 0.000 is benign and 1.000 is damaging. MutationTaster: the probability value refers to the prediction, that is, a value close to 1 indicates a high ‘‘security’’ of the prediction. SNP: single nucleotide polymorphisms.

Supplementary Table 2. CRISPR sgRNA sequences used for inactivation of *METTL3* and *YTHDF2* genes.

| Gene Targeted | sgRNA Sequence (5’-3’) |
| --- | --- |
| *METTL3* | GGACACGTGGAGCTCTATCC |
| *YTHDF2* | GGGTGAAGCTGCTTGGTCTAC |

Supplementary Table 3. Sanger sequencing of gene knockout PCR primers

| **Name** | **Forward primer 5’-3’** | **Reverse primer5’-3’** |
| --- | --- | --- |
| *METTL3* | ATGAGAGAGCGGGTATGAGGACTAT | GGTAAAAGTGAGCTGGAAGAACCGA |
| *YTHDF2* | GTGCTAAAGCTAAGGCCCATATTCA | AATGGAGTGCTACCTAGGGC |

Supplementary Table 4. RT-qPCR and clone primers

| **Name** | **Forward primer 5’-3’** | **Reverse primer5’-3’** |
| --- | --- | --- |
| *GAPDH* | CGCTTCGCTCTCTGCTCCTCCTGT | GGTGACCAGGCGCCCAATACGA |
| *BLIMP1* | CGGGGAGAATGTGGACTGGGTAGAG | CTGGAGTTACACTTGGGGGCAGC |
| *PLZF* | GAGATCCTCTTCCACCGCAAT | CCGCATACAGCAGGTCATC |
| *NANOS3* | CCCGAAACTCGGCAGGCAAGA | AAGGCTCAGACTTCCCGGCAC |
| *TFAP2C* | CGCTCATGTGACTCTCCTGACATCC | TGGGCCGCCAATAGCATGTTCT |
| *POU5F1* | GCTGGAGCAAAACCCGGAGG | TCGGCCTGTGTATATCCCAGGGTG |
| *SOX2* | ACACCAA TCCCA TCCACACT | CCTCCCCAGGTTTTCTCTGT |
| *SOX17* | GAGCCAAGGGCGAGTCCCGTA | CCTTCCACGACTTGCCCAGCAT |
| *VASA* | TTCTTCACAAGCTCCCAATCCA | TTCTTCTCTGCATCAAAACCACA |
| *ID4* | TCCCGCCCAACAAGAAAGTC | TGTCGCCCTGCTTGTTCAC |
| *NANOG* | AGAGGTCTCGTATTTGCTGCAT | AAACACTCGGTGAAATCAGGGT |
| *DMRT3* | ATGTGGCAAAGAGTAAGGGCT | GCGGTCTGTTGGCTTTCAAG |
| *GFRa1* | CCAAGCACAGCTACGGAATG | CAGGCACGATGGTCTGTCG |
| *LINE-1* | CAAACACCGCATATTCTCACTCA | CTTCCTGTGTCCATGTGATCTCA |
| *NANOS2* | TGTGGAAGGACTACTTCAACCT | GGGACTTGGCTCCTCAATCT |
| *PIWIL4* | CTTGTGGACAACATCCAGAGGAATAC | CACAATCCGGCCAGTCAGAGA |
| *β-actin* | GAAAATCTGGCACCACACCT | GATAGCACAGCCTGGATAGCAA |
| *METTL3* | TCTGGGGGTATGAACGGGTA | CTGGTTGAAGCCTTGGGGAT |
| *L1-5′UTR clone* | gctgggtaccagctgGGGGAGGAGCCAAGATGG | cccatctttgtggttttagCTTTGTGGTTTTATCTACTTTTGGTCTTTG |

Supplementary Table 5. MeRIP-qPCR and RIP-qPCR primers

| **Name** | **Forward primer 5’-3’** | **Reverse primer5’-3’** |
| --- | --- | --- |
| *L1-5′UTR* | AGCCTAACTGGGAGGCA | TTCCCATCTTTGTGGT |
| *L1-ORF1p* | GAATGATTTTGACGAGCTGAGAGAA | GTCCTCCCGTAGCTCAGAGTAATT |
| *NANOG* | AGAGGTCTCGTATTTGCTGCAT | AAACACTCGGTGAAATCAGGGT |

Supplementary Table 6. Antibodies used in this study

| **Antibody** | **Company** | **Cat NO.** |
| --- | --- | --- |
| POU5F1 | BD Biosciences | 560589 |
| NANOG | BD Biosciences | 560589 |
| SOX2 | BD Biosciences | 560589 |
| CD90 | BD Biosciences | 555595 |
| CD24 | BD Biosciences | 560991 |
| PLZF | Thermo Fisher | 12-9320-80 |
| PLZF | SantaCruz Biotechnology | sc-28319 |
| VASA | SantaCruz Biotechnology | sc-517247 |
| GPR125 | abcam | ab51705 |
| YTHDF2 | abcam | ab246514 |
| G3BP1 | abcam | ab56574 |
| m6A | Synaptic Systems | 202003 |
| LC3B | ABclonal | A17424 |
| ORF1p | abcam | ab216324 |
| p62 | ABclonal | A19700 |
| METTL3 | abcam | ab195352 |
| WTAP | abcam | ab195380 |

Supplementary Table 7. RNA-seq result for all samples
